# A high confidence, manually validated human blood plasma protein reference set

**DOI:** 10.1186/1755-8794-1-41

**Published:** 2008-09-15

**Authors:** Susann Schenk, Gary J Schoenhals, Gustavo de Souza, Matthias Mann

**Affiliations:** 1 Department of Biochemistry and Molecular Biology, Bioinformatics, University of Southern Denmark, Campusvej 55, 5230 Odense M, Denmark; 2 University of Bergen, P.O. Box 7800, 5020 Bergen, Norway; 3 Proteomics and signal transduction, Max-Planck Institute for Biochemistry, Am Klopferspitz 18, 81152 Martinsried, Germany

## Abstract

**Background:**

The immense diagnostic potential of human plasma has prompted great interest and effort in cataloging its contents, exemplified by the Human Proteome Organization (HUPO) Plasma Proteome Project (PPP) pilot project. Due to challenges in obtaining a reliable blood plasma protein list, HUPO later re-analysed their own original dataset with a more stringent statistical treatment that resulted in a much reduced list of high confidence (at least 95%) proteins compared with their original findings. In order to facilitate the discovery of novel biomarkers in the future and to realize the full diagnostic potential of blood plasma, we feel that there is still a need for an ultra-high confidence reference list (at least 99% confidence) of blood plasma proteins.

**Methods:**

To address the complexity and dynamic protein concentration range of the plasma proteome, we employed a linear ion-trap-Fourier transform (LTQ-FT) and a linear ion trap-Orbitrap (LTQ-Orbitrap) for mass spectrometry (MS) analysis. Both instruments allow the measurement of peptide masses in the low ppm range. Furthermore, we employed a statistical score that allows database peptide identification searching using the products of two consecutive stages of tandem mass spectrometry (MS3). The combination of MS3 with very high mass accuracy in the parent peptide allows peptide identification with orders of magnitude more confidence than that typically achieved.

**Results:**

Herein we established a high confidence set of 697 blood plasma proteins and achieved a high 'average sequence coverage' of more than 14 peptides per protein and a median of 6 peptides per protein. All proteins annotated as belonging to the immunoglobulin family as well as all hypothetical proteins whose peptides completely matched immunoglobulin sequences were excluded from this protein list. We also compared the results of using two high-end MS instruments as well as the use of various peptide and protein separation approaches. Furthermore, we characterized the plasma proteins using cellular localization information, as well as comparing our list of proteins to data from other sources, including the HUPO PPP dataset.

**Conclusion:**

Superior instrumentation combined with rigorous validation criteria gave rise to a set of 697 plasma proteins in which we have very high confidence, demonstrated by an exceptionally low false peptide identification rate of 0.29%.

## Background

Human blood plasma contains a plethora of proteins, encompassing not only proteins that have plasma-based functionality, but possibly every other human protein in minute amounts as well. Circulating through the tissues, the plasma picks up proteins that are released from their origin due to physiological events such as tissue remodeling and cell death. Specific disease processes or tumors are often characterized by plasma "signatures", which may become obvious via changes in the plasma proteome profile, for example, through over-expression of proteins.

Thus, besides being a medically relevant diagnostic tool, the plasma is also of exceptional nature, characterized by its complexity and its large dynamic protein concentration range. Additionally, because of the potential for every possible human protein to be present, there is an inherent difficulty in distinguishing between proteins truly residing in the plasma and proteins that are released into the plasma due to trauma or other events. Fortunately, it is to be expected that the latter are found inconsistently and usually only in very low concentration, below the limits of detection.

Thirty years ago the detection of plasma proteins became feasible with the introduction of two-dimensional (2D)-gel electrophoresis, but the analysis of unfractionated plasma substantially limited the number of detectable proteins, resulting in a total of only 60 identified plasma proteins by 1992 [1]. The combination of 2D-gel electrophoresis, removal of the most abundant serum proteins with immunoaffinity chromatography, and sequential anion-exchange and size-exclusion chromatography, and subsequent MALDI-TOF as well as online electrospray ion trap mass spectrometry, increased the number of distinct plasma proteins identified to 325 eleven years later [2]. Progressively more proteins could be identified as technological advancements were introduced and different preparative techniques were combined. The pilot phase of the PPP, launched by HUPO in 2002, attempted to address questions regarding the best technology platform for the characterization of proteins in human plasma or serum. The PPP investigated factors such as the influence of various technical aspects of specimen collection and handling, whether the most abundant plasma proteins should be depleted, and whether anti-protease cocktails are desirable [3,4]. In the end, 35 proteomics laboratories in 13 countries committed to participate in the PPP. Most of the laboratories separated their samples at the peptide level using liquid chromatography, followed by MALDI- or electrospray-MS2. The software used for peptide identification included Sequest, Mascot, PepMiner, Viper, Digger, and Sonar. Several investigators applied combinations of these technologies. The bioinformatics group at the University of Michigan was the central hub of the project, being responsible for validating the submitted protein identifications [5].

To complement the efforts of the PPP, we established a reference set of plasma proteins that we are highly confident in and against which other data sets can be compared. As a single lab, we clearly could not address all of the possible technical variables addressed by the PPP. For example, we decided to use human plasma rather than serum in order to avoid any *in vitro *proteolysis processes which may have introduced artifacts. However, in parallel with the PPP effort, we employed depletion and pre-fractionation methods to deal with the enormous complexity and plasma protein concentration range, and we also used protease inhibitors. Comparison of the various techniques employed revealed the usefulness of some of those techniques. We utilized hybrid LTQ-FT and -Orbitrap mass spectrometer systems for plasma measurements because of their superior dynamic range and mass accuracy, and to further increase the reliability of our data, we employed MS3.

## Methods

### Plasma sample preparation and protein depletion

Blood samples analysed by FT-ICR were drawn from two healthy male volunteers aged 39 and 46, pooled, mixed with EDTA to prevent blood clotting, and kept on ice until being centrifuged at 400 × g for 20 min at 4°C. If plasma samples were not immediately used for analysis, they were stored at -80°C until needed. To remove albumin from plasma, the Vivapure anti-HSA kit (VivaScience, Hannover, Germany) was used according to the instructions of the manufacturer. Protease inhibitors were added to selected samples before albumin depletion using Protease Inhibitor Cocktail tablets (Complete, Roche Diagnostics, Penzberg, Germany). Depletion of 6 of the most abundant proteins including albumin, transferrin, haptoglobin, alpha-1-antitrypsin, IgA and IgG was performed using the Agilent Multiple Affinity Removal System (Agilent Technologies, Waldbronn, Germany). Blood samples to be subjected to the Orbitrap were collected and pooled from 9 healthy individuals, 5 males (aged 35–56 years) and 4 females (aged 26 to 40 years) with no family history of diabetes or blood disorders. EDTA was added at sample collection time, protease inhibitors were later added and high abundance proteins were removed using the Agilent Multiple Affinity Removal System described above.

### Gel electrophoresis and protein digestion

Gel electrophoresis was performed with pre-cast NuPage Bis-Tris gels (4–12%) and MES or MOPS buffer (Invitrogen, Carlsbad, CA) according to the manufacturer's instructions. Alternatively, plasma samples were run on large 4–20% Tris-glycine gradient gels. Gels were stained with a Colloidal Blue Staining Kit (Invitrogen). Protein bands were excised, cut into small pieces and washed at least twice (20 min. each) with 50:50 (v/v) 50 mM NH_4_HCO_3_/absolute HPLC-grade ethanol. Supernatants were discarded after each washing step. The gel pieces were dehydrated with absolute ethanol until opaque, white and hard. Disulfide bonds were cleaved with 10 mM DTT in 50 mM NH_4_HCO_3 _buffer (not pH adjusted) for 60 min at 56°C. Alkylation of cysteines was performed by the addition of 55 mM iodoacteamide in 50 mM NH_4_HCO_3 _buffer and incubation of the samples for 45 min at room temperature in the dark. Gel pieces were washed twice in 50 mM NH_4_HCO_3 _buffer and dehydrated with absolute ethanol, dried (Speed-Vac) and covered with trypsin (modified sequence grade, Promega, Madison, WI, U.S.A.) solution (12.5 ng/μl trypsin in 50 mM NH_4_HCO_3_). Protein digestion was performed at 37°C overnight and stopped by the addition of a final concentration of 3% (v/v) trifluoroacetic acid (TFA). Supernatants were collected and gel slices were extracted at least twice with 100% acteonitrile. Supernatants were pooled and the acetonitrile was removed using Speed-Vac centrifugation. Samples were acidified with TFA to pH ≤ 2.5, loaded on conditioned C-18 tips, and stored at 4°C until used for mass spectrometry.

Plasma sample separation by off-gel electrophoresis (OGE) with subsequent 2-D gel electrophoresis for separation evaluation was performed at Agilent (Waldbronn, Germany). OGE fractions were treated in solution with iodoacetamide, subsequently quenched with DTT and concentrated using Microsep columns (molecular weight cut-off (MWCO) 3 kDa, PALL Life Sciences, Ann Arbor, MI, USA). Trypsin digestion, disulfide reduction and cysteine alkylation were performed as specified above.

### Nano-HPLC and mass spectrometry

Nanoscale liquid chromatography tandem mass spectrometry (nano-HPLC-MS/MS) was performed using an Agilent 1100 nanoflow LC system (Agilent Technologies), equipped with a solvent degasser, a nanoflow pump and a thermostatted autosampler. This system was connected to a 7 Tesla Finnegan linear quadrupole ion trap Fourier transform (LTQ-FT) mass spectrometer and an LTQ-Orbitrap (Thermo Electron, Bremen, Germany). Tryptic peptides were chromatographically separated on 15 cm columns (75 μm inner diameter) packed by hand with a methanol-based slurry of reverse-phase ReproSil-Pur C18-AQ 3 μm resin (Dr. Maisch HPLC GmbH; Ammerbuch, Germany) and mounted on the nanoelectrospray ion source. Peptides were autosampled onto the packed column at a flow rate of 500 nl/min and were separated over 20 min using a linear gradient of 13–34% (v/v) acetonitrile/0.5% (v/v) acetic acid. Elution occurred at a flow rate of 250 nl/min and ionization was performed using an applied voltage of 2.4 kV to the emitter.

Data were acquired using Xcalibur software in data-dependent mode to facilitate automatic switching between MS, MS2 and MS3. In the case of LTQ-FTICR survey, full scan MS spectra (from m/z 300–1,575) were acquired in the ICR with a resolution R = 25,000 at m/z 400 (after accumulation to a target value of 5 × 10^6 ^in the linear ion trap). The three most intense ions were sequentially isolated for accurate mass measurements by FT ICR selected ion monitoring [5] scan with 10 Da mass range, R = 50,000 and target accumulation value of 5 × 10^4 ^ions. Simultaneously, these were fragmented in the linear ion trap by collision-induced dissociation (MS2) at a target value of 5 × 10^4 ^ions. For MS3 analysis the three most intense ions of each MS2 spectrum with m/z > 300 were further isolated and fragmented. Each precursor ion selected for MS2 was dynamically excluded for 30 s for subsequent LC-MS runs. Total cycle time was approximately 3 s. General mass spectrometric conditions were as follows: no sheath and auxiliary gas flow, ion transfer tube temperature 100°C, collision gas pressure 1.3 mTorr, normalized collision energy using wide band activation mode was 30% for MS2 and 35% for MS3, ion selection thresholds were 250 counts for MS2 and 5 counts for MS3. An activation q = 0.25 and activation time of 30 ms was applied in both MS2 and MS3 acquisition. In the case of the LTQ-Orbitrap, the precursor ion scan MS spectra (m/z 300–1600) were acquired in the Orbitrap with resolution R = 60,000 at m/z 400 with the number of accumulated ions being 1 × 10^6^. The five most intense ions were isolated and fragmented in the linear ion trap (number of accumulated ions; 3 × 10^4^). The resulting fragment ions were recorded in the Orbitrap with resolution R = 15,000 at m/z 400. The lock mass option enabled accurate mass measurements in both MS and MS/MS mode. The polydimethylcyclosiloxane ions generated in the electrospray process from ambient air (protonated (Si(CH_3_)_2_O)_6_, m/z 445.120025) were used for internal recalibration in real time. In data-dependent LC-MS/MS experiments dynamic exclusion was used with 30 s exclusion duration.

### Database searches

Using software available in-house (DTA Supercharge), all MS/MS spectrum files from each LC run were converted into peak-lists. The charges and error masses were also assigned, and files were centroided and merged into a single file. These were searched against the human IPI (International Protein Index) database (versions 2.27, 2.35, 2.37, 3.03, 3.19; all versions were later converted to version 3.25) using the Mascot search engine (Matrix Science, London, UK) with carbamidomethyl cysteine as a fixed modification and variable modifications, including oxidized methionine (+15.99 Da), protein N-acetylaction, deamidation [6], and Pyro (N-term QEC). Searches were done with tryptic specificity allowing one missed cleavage and a tolerance on mass accuracy of 5 ppm in MS mode and 0.5 Da in MS/MS mode. MS3 spectra were automatically scored using MSQuant software [7] according to an algorithm that assigns MS3 spectra to peptide fragment sequences [8]. MSQuant is a validation tool developed in-house that parses Mascot identifications and allows for MS3 scoring, quantitation, and manual spectrum verification.

### Blood plasma protein database (BPPD) construction and associated software

Identified proteins were exported from MSQuant along with their appropriate sequenced peptides, all of their assigned accession numbers, Mascot peptide scores, MS3 precursor ions and other MS and MS/MS relevant data into Microsoft Excel before being uploaded into a blood plasma protein database. The BPPD was built in-house using the open source relational database MySQL [9]. The associated web-based software tools were written in Perl [10]. The BPPD architecture stores all of the peptides for each protein identified, the associated protein information (including the non-redundant protein and peptide sequences), as well as the mass spectrometry data associated with each peptide. Experimental design data is also included to make cross-experiment comparisons possible.

To establish a 'finalized' set of proteins, stringent criteria were applied with regard to peptide length and peptide Mascot and MS3 scores (see Results). Note that we have decided to exclude proteins belonging to the immunoglobulin family in order to facilitate comparison with other blood plasma studies, but we have included the data for these proteins in a supplement available on line [see Additional file 1]. To aid in the data validation process, several web-based software tools were developed in-house, such as a tool to parse and reorganize the output from MSQuant, a tool to retrieve SwissProt and NCBI sequences and their respective annotations based on accession numbers, as well as a 'peptide mapper', which allows the mapping of peptides onto sequences in plain text, FASTA, or ClustalW sequence alignment format. We also developed other software tools for the purpose of identifying redundancy, automating the peptide validation process and for calculating the percent peptide coverage. A detailed description of the software tools and database system used here will be published elsewhere.

### Decoy database search to estimate false peptide identification rate

In order to estimate the rate of false peptide identification in our result set, we constructed a decoy database consisting of all of the sequences of the human IPI database (version 3.25; 67,250 sequences) in their reverse orientation, together with the original FASTA header information. 'Reverse orientation' simply means that each protein sequence was read and stored sequentially, beginning at the C-terminal end and concluding at the N-terminal end. We then extracted all of the non-redundant peptide sequences obtained for all of the experiments we performed and searched with these against the decoy database that we constructed. The false error rate was reported as the number of peptide sequences matching the decoy database divided by the total number of non-redundant peptides we obtained across all experiments. This number was then converted to a percentage [11,12].

## Results

### Plasma experiments performed

In order to identify a set of plasma proteins with high confidence and to test the usefulness of different plasma treatments and separation methods, we employed several different techniques: depletion of highly abundant proteins, addition of protease inhibitors, two different pre-fractionation methods, as well as modified mass spectrometry settings. We performed a total of eight different independent experiments (Table 1).

**Table 1:** Plasma experiments performed.

Experiment	Designated name	Plasma treatment	[Protein] (μg) applied	Plasma separation method	MS	LC-MS run time/sample (minutes)	Comment
Plasma_01	01_MS2	none	450	1-D PAGE	MS/MS2	100	
Plasma_04	04_MS2_prec	none	750	1-D PAGE	MS/MS2	100	Precursor selection within certain mass ranges only
Plasma_05	05	none	750	1-D PAGE	MS/MS2/MS3	100	
Plasma_06	06_Alb_depl	Albumin-depletion	1800	1-D PAGE	MS/MS2/MS3	100	
Plasma_10	10_Alb_depl _NL	Albumin depletion, Protease inhibitors	1800	1-D PAGE	MS/MS2/MS3	100	Neutral loss dependent MS3
Plasma_08	08_OGE	none	1800	OGE	MS/MS2/MS3	140	
Plasma_09	09_OGE_6_depl	Depletion of albumin, transferrin, haptoglobin, alpha-1-antitrypsin, IgA and IgG	650	OGE	MS/MS2/MS3	140	
Plasma_11	11_Orbitrap	Depletion of albumin, transferrin, haptoglobin, alpha-1-antitrypsin, IgA and IgG	300	1-D PAGE	MS/MS2	140	

A summary of the differences in the experimental protocols between plasma experiments is shown. Each experiment has been assigned a meaningful "designated name" that is used in the text and in the accompanying figures and tables. Note that all experiments include MS3 measurements with the exception of 01_MS2, 04_MS2_prec and 11_Orbitrap. The type of treatment performed on the plasma samples, if any, is indicated, along with the amount of material applied to the gel, the separation method employed, and the method used for MS data collection.

For experiments 01_MS2 and 04_MS2_prec, plasma was separated on a Novex 4–12% pre-cast gradient gel (gel size 8 × 8 cm) and a larger 4–20% manually poured gradient gel (gel size 12.5 × 14 cm), respectively. The Novex gel was cut into 29 pieces, and about 50% of the larger gel into 21 pieces. The size of the gel slices was chosen individually for each gel depending on the protein intensity and band size. MS and MS2 spectra were recorded in a total LC-MS run time of 100 min for each of the 29 and 21 samples from 01_MS2 and 04_MS2_prec, respectively. For 01_MS2 samples, scan events were performed as described in Methods 'HPLC and Mass Spectrometry', except that MS3 was not recorded (MS full scan, m/z 300.0 – 1500.0). For 04_MS2_prec the acquisition software (MS full scan, m/z 300.0–1800.0) was directed to select only peptides in the amplified mass ranges for sequencing (m/z 350.0–450.0, 445.0–545.0, 540.0–640.0, 635.0–735.0, 730.0–830.0, 825.0–1800.0). The 05 experiment is similar to 04_MS2_prec with respect to the protein amount but the gel was cut into 35 fractions. Also, MS3 was performed in addition to MS and MS2, and MS scan events were carried out as described in Methods 'nano-HPLC and mass spectrometry' (MS full scan, m/z 300.0–1575.0). The MS run time was 100 min per sample. Compared to 05, approximately three times more plasma protein was employed and was albumin-depleted before gel separation in the 06_Alb_depl experiment. The gel was cut into 28 pieces and the MS settings were basically the same for 05 and 06_Alb_depl. The addition of Protease Inhibitors distinguishes experiment 10_Alb_depl_NL from 06_Alb_depl. Also, in 10_Alb_depl_NL, MS3 acquisition was only done in a neutral loss-dependent fashion, in order to detect possible phosphopeptides. As early as in the pilot phase of the HUPO project [3-5], the possible determination of posttranslational modifications of plasma proteins such as protein phosphorylation was mentioned as an important issue for the comprehensive analysis of the protein constituents of human plasma as well as the identification of biomarkers. Finally, in the 08_OGE and 09_OGE_6_depl experiments, plasma proteins were not separated by 1D-PAGE, but by off gel electrophoresis (OGE). For experiment 09_OGE_6_depl, 12 mg of plasma was applied to the Agilent Multiple Affinity Removal System (removal of 6 high abundance proteins) and 50% of the depleted protein (650 ug) was subsequently separated by OGE. For experiment 08_OGE, plasma was directly applied to OGE. 15 fractions from each experiment were subjected to mass spectrometry, which was performed in essentially the same manner as mentioned for 05 and 06_Alb_depl, except that the total run time was increased to 140 min per fraction sample. Plasma samples that were designated for measurement on the Orbitrap were depleted of 6 high abundance proteins as above and run in 3 separate lanes on a 1D-PAGE gel. Each lane was cut into 15 slices resulting in a total of 45 samples. The samples were analysed separately on the Orbitrap without MS3 analysis.

### Data validation and blood plasma protein database

The mass spectrometry LTQ-FT acquisition of 7 plasma samples was designed to exploit the sensitivity and speed advantages of the ion trap, while taking advantage of the ultra-high mass accuracy and dynamic range of the Fourier Transform ion cyclotron resonance (FT-ICR) detector. The Orbitrap mass analyser features very high sensitivity in MS and MSn and rapid scan rates. Its excellent mass accuracy capabilities and high resolution are similar to those achievable with FT-ICR instrumentation.

In total, protein and peptide identifications from 8 independent experiments corresponding to 216 MS runs, were combined within our Blood Plasma Protein Database (BPPD). In addition to the stringent Mascot search criteria (tryptic specificity, 1 missed cleavage, MS accuracy of 5 ppm and MS2 of 0.5 Da), only peptides that were checked (highest scoring use of spectra), red (highest scoring match of spectra), bolded (first use of spectra in the output list), and had a minimum Mascot peptide score of 16 as well as a minimum length of 7 amino acids were considered for further validation.

Further validation criteria were applied as previously described [13,14] and as defined by Mascot peptide identification/assignment software (Matrix Science, London, UK). Briefly, proteins identified with one single peptide were required to have an MS3 spectrum, an MS3 score, and a total score (also known as a 'Mascot peptide score plus MS3 score') ≥ 42, which assured with 99.9% confidence that this was a correct identification. Proteins identified with one single peptide but without an available MS3 scan were discarded regardless of their Mascot peptide score. If a protein was identified with two peptides, one of the two peptides was required to have a Mascot peptide score ≥ 32, ensuring a minimum confidence of 99%, and the other peptide a 95% confidence (Mascot peptide score ≥ 25) of being a correct identification. Proteins identified with three peptides were required to have at least one peptide with a Mascot peptide score ≥ 32 (99% confidence). In order to automate the validation process, a Perl script was written that applied the validation rules stated above, and which also verified that each and every peptide sequence actually mapped correctly to the master protein sequence.

All proteins seemingly identified as a specific isoform were manually verified. Using ClustalW and our 'peptide mapper' software, the peptides for a particular protein were matched to all known isoforms for this protein, and in the case where one or more of the peptides was specific for one and only one single isoform, this protein was given the specific protein isoform name. If the peptides mapped onto more than one isoform then all possible isoforms were added to the annotation for that protein, but not counted as separate identified proteins.

Additionally, all proteins were assigned a primary accession number; that is, an accession number and sequence respectively (Swiss-Prot, Tremble, RefSeq, Ensembl or H-Inv) that matched all identified peptides for a given protein. If the peptides for a given protein matched more than one accession number/sequence, then the Swiss-Prot/Uni-Prot number was chosen, if available.

Several checks to eliminate redundancy from the BPPD were performed. First, all proteins with identical MW and/or overlapping accession numbers were manually verified using our peptide mapping software. We encountered a substantial number of overlapping accession numbers since Mascot provides accession number information from many sources. In these cases all of the peptides from all of the proteins in question were 'mapped' to all of the possible protein sequences. Note that to say that a peptide 'maps to a sequence', we required a 100% sequence match between the peptide(s) and the protein sequence(s) in question. If the peptides from all of the overlapping proteins were found to completely map to one of the protein sequences, then the data was merged into one entry and the protein name that is given in the SwissProt/Uniprot database was chosen as the annotation for that entry. If even one peptide failed to map during this test then that protein remained as a separate entry in the database.

In cases where the Mascot-assigned peptides of a protein did not match any assigned accession number/sequence, then the peptides were used to perform a BLAST search for short, nearly exact matches. If there was no unambiguous identification possible following this step then the protein and peptides were discarded.

After removal of the first set of redundant entries, all remaining entries in the BPPD were aligned to each other using All *vs. *All BLAST [15] and all alignments with 90% or higher identity were again manually checked for redundancy using a ClustalW alignment and our peptide mapping software.

As a final redundancy check, we performed a database search with the aid of a Perl script we developed which employs a peptide mapping approach to identify redundancy. We located redundancy by identifying protein entries whose peptides also completely mapped to other protein entries. In these cases we reassigned the peptides belonging to the redundant protein entries to one common master protein entry. During this stage we were somewhat surprised to discover that not all instances of redundancy for highly homologous proteins were detected in the all *vs*. all BLAST step that we carried out previously. For example, isoforms 2 and 4 of Fibronectin 1 were not identified as redundant using the All vs. All BLAST tool with a cutoff of 90% identity due to a poor alignment of the sequences by the BLAST program. The isoform 2 and 4 sequences of Fibronectin 1 share 100% sequence identity, except for a central region within isoform 2 which makes this isoform 91 amino acids longer than isoform 4. In retrospect, employment of the peptide mapping approach as the first step rather than as the last step in redundancy checking would probably have rendered all of the other redundancy checks that we performed unnecessary.

Our elaborate validation process is reflected in the results of the decoy database analysis in which we searched all of the human International Protein Index (IPI) sequences (67,250) in their reversed orientation, which yielded 30 matches out of a possible 10,378. This gave a false peptide identification rate of 0.29 percent. In this analysis, the possible peptides (10,018) for the search were calculated by assembling all of the peptide sequences across all experiments and removing all of the peptides identifying immunoglobulins. Redundancy among the peptide sequences was then eliminated. The number of 10,018 peptides differs from those seen below in that it includes the peptides for all proteins (excluding immunoglobulins) across all experiments prior to validation.

### High confidence set of plasma proteins

**Figure 1:**
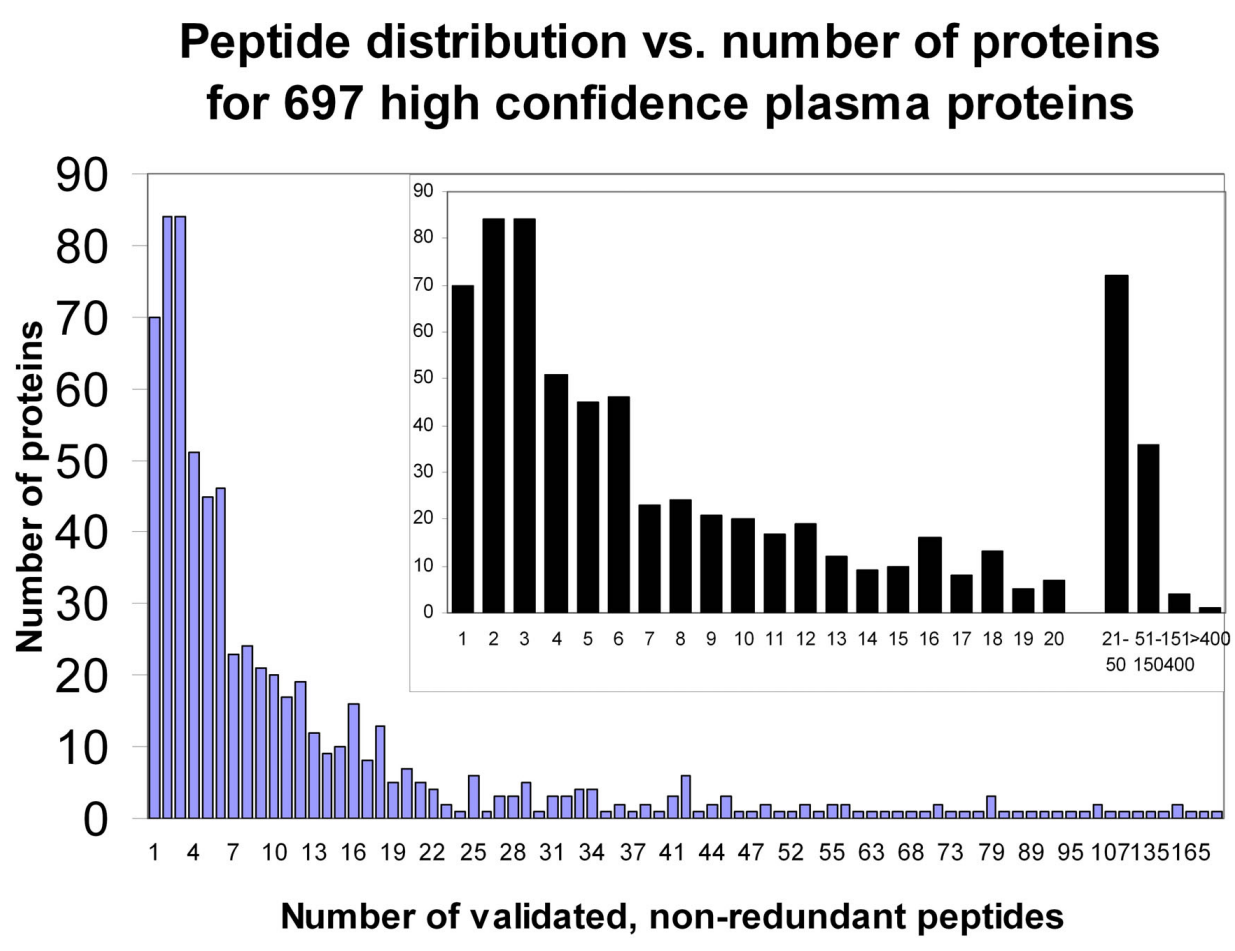
**Histogram showing the number of proteins identified *versus *the number of validated, non-redundant peptides found for each protein, across all experiments**. The number of validated, non-redundant peptides used to identify each protein was calculated and the proteins with identical numbers of peptides were plotted in the same group, indicated on the X-axis. For example, there are a total of 70 proteins that were identified with a single peptide. The inset depicts in detail the number of proteins that were identified with 1–20 peptides. Proteins identified with more than 20 peptides were categorized into groups as indicated.

By combining the eight plasma experiments as listed in Table 1 and after removal of redundancy, we identified 1193 distinct proteins with a valid primary accession number. The combination of rigorous data validation coupled with the removal of immunoglobulin-related proteins for separate analysis reduced this number to 697, which we refer to as our 'stringently validated high confidence protein set'. 70 of these 697 proteins were identified with 1 peptide (Figure 1) and 84 proteins were identified with exactly 2 peptides. The remaining 541 proteins were identified with 3 or more peptides. 314 proteins were identified with 3–10 peptides and 229 proteins had 11 or more peptides. 12 proteins were identified with over 100 peptides (Table 2). The protein identified with the highest number of peptides was apolipoprotein B-100 (505 validated non-redundant peptides), while albumin, a protein known to be extremely abundant in blood plasma, was identified with 94 validated, non-redundant peptides.

**Table 2:** Proteins identified with more than 100 non-redundant, validated peptides.

Protein name	Number of distinct, validated peptides
Apolipoprotein B-100 [Precursor]	505
Complement C3 [Precursor] variant	235
Complement C3 [Precursor]	165
Alpha-2-macroglobulin [Precursor]	164
Complement component C4B, C4B1	164
Fibronectin [Precursor], isoform 1, 3, 5, 7, 8, 9, or 10	143
Talin-1	135
Filamin A, alpha (actin binding protein 280)	117
Fibronectin 1, isoforms 3, 4 or 5, or CRA isoforms h, j, n or m	115
Fibronectin [Precursor], isoform 8	107
Complement component 4A	105
Complement C5 [Precursor]	105

After combining the experimental data for all 8 treatments described here, the number of validated, non-redundant peptides was calculated for each protein and the proteins having 100 or more such peptides appear in this list. The list is arranged in descending order beginning with the protein having the largest number of distinct, validated peptides.

The 697 validated proteins in our list [see Additional file 2] were identified with 37,682 non-validated, redundant peptides. This was calculated by summing all of the redundant peptides for each validated protein in our list across all experiments. Of these peptides, 246 did not pass our validation criteria. Note that peptides that were invalidated and which belonged to proteins that ultimately failed validation were not included in this number. Our list of 697 validated proteins was thus identified with 37,436 validated, redundant peptides. Following removal of peptide redundancy on a per protein basis, our list of 697 validated proteins was identified with 10,145 validated, non-redundant peptides, which equates to an average of almost 14.6 validated, non-redundant peptides per protein and a median of 6 non-redundant peptides per protein. The number of validated, non-redundant peptides was calculated by considering each protein separately. The redundancy within each protein's peptides was removed and the remaining peptides were summed. Removal of the redundancy after pooling the peptides from all validated proteins left us with 9263 valid, non-redundant peptides. As mentioned, these numbers refer to the dataset excluding all proteins annotated as immunoglobulins and proteins whose peptides mapped completely to immunoglobulin protein sequences but were annotated as hypothetical proteins. We did not comprehensively examine the similarity of all protein sequences from our validated list to immunoglobulin sequences.

**Figure 2:**
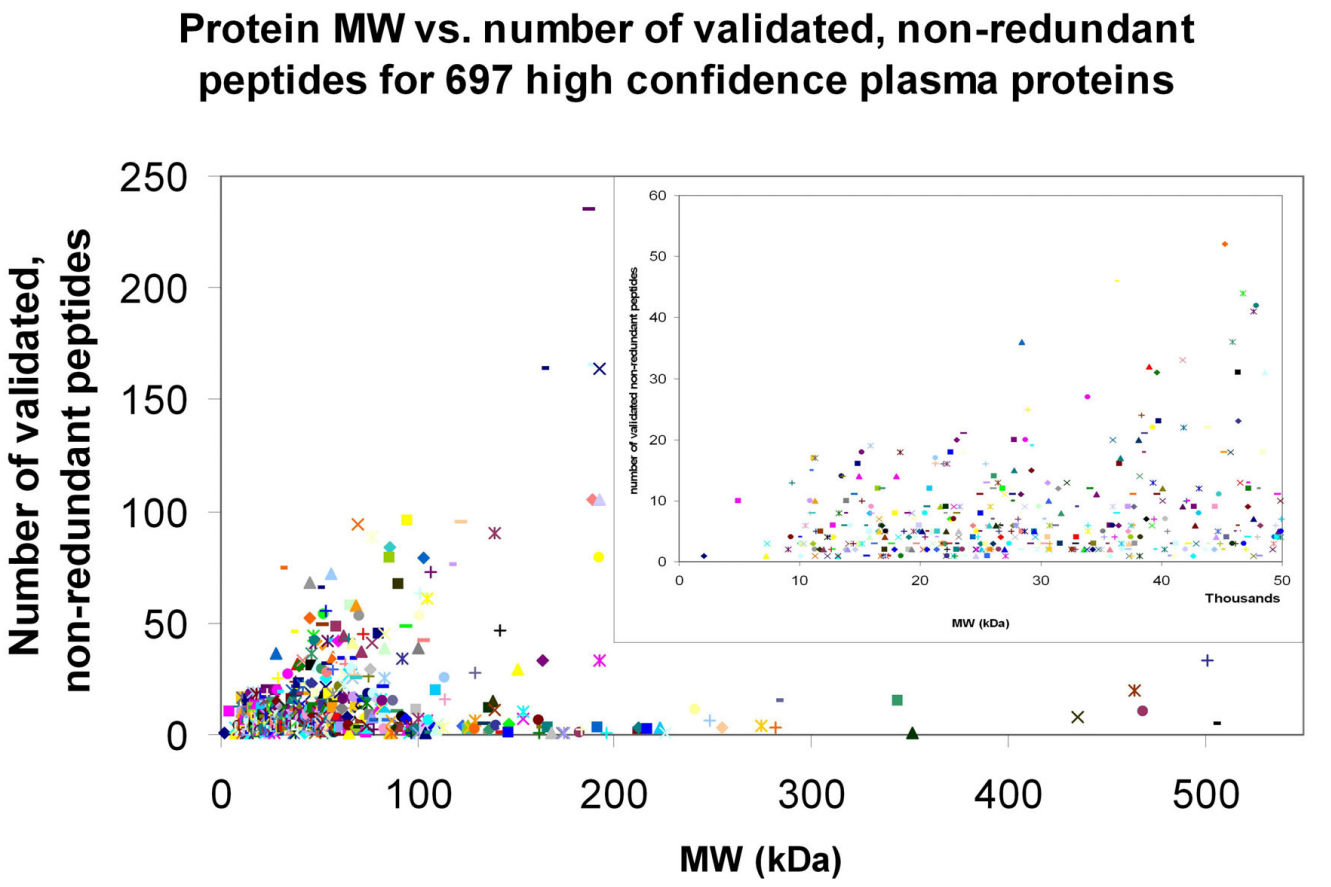
**Histogram depicting the number of validated, non-redundant peptides versus the MW of the identified proteins**. The number of validated, non-redundant peptides used to identify each protein was calculated and this number was plotted as a function of the molecular weight of that particular protein. The MW range (X-axis) was truncated at 550 kDa, resulting in the loss of one protein. Likewise, the number of validated, non-redundant peptides (Y-axis) was truncated at 250 peptides, resulting in the loss of an additional protein.

If we depict the number of validated non-redundant peptides *versus *the MW of the appropriate protein, it is very clear that most proteins identified have a MW below 100 kDa and not more than 50 unique peptides (Figure 2). As expected, smaller proteins tend to be identified with fewer peptides than larger proteins. It appears that 50 peptides are, in general, the maximum number of peptides sequenced, even for larger proteins.

From a total of 37,682 non-validated, redundant peptides, the highest proportion of peptides (3971) had a length of 9 amino acids, followed by 3487 peptides with 11 amino acids, 3401 peptides with 10 and 3149 peptides with 8 amino acids in length. The longest peptides sequenced were 59 amino acids in length. 995 of the 10,145 validated, non-redundant peptides were 9 amino acids in length, followed by 856 peptides with 10, and 852 peptides with a length of 11 amino acids. We identified a total of 749 validated, non-redundant peptides having a length of 7 amino acids, which comprised approximately 7.4% of 10,145 validated, non-redundant peptides in total for the set of 697 high-confidence proteins. 285 of the 697 proteins were identified with a set of peptides among which we found at least one peptide with a length of 7 amino acids. 324 and 365 of the 697 proteins were identified with a set of peptides that included peptides with 8 and 9 amino acids in length respectively. 184 proteins were exclusively identified with peptides ≥ 10 amino acids in length.

**Figure 3:**
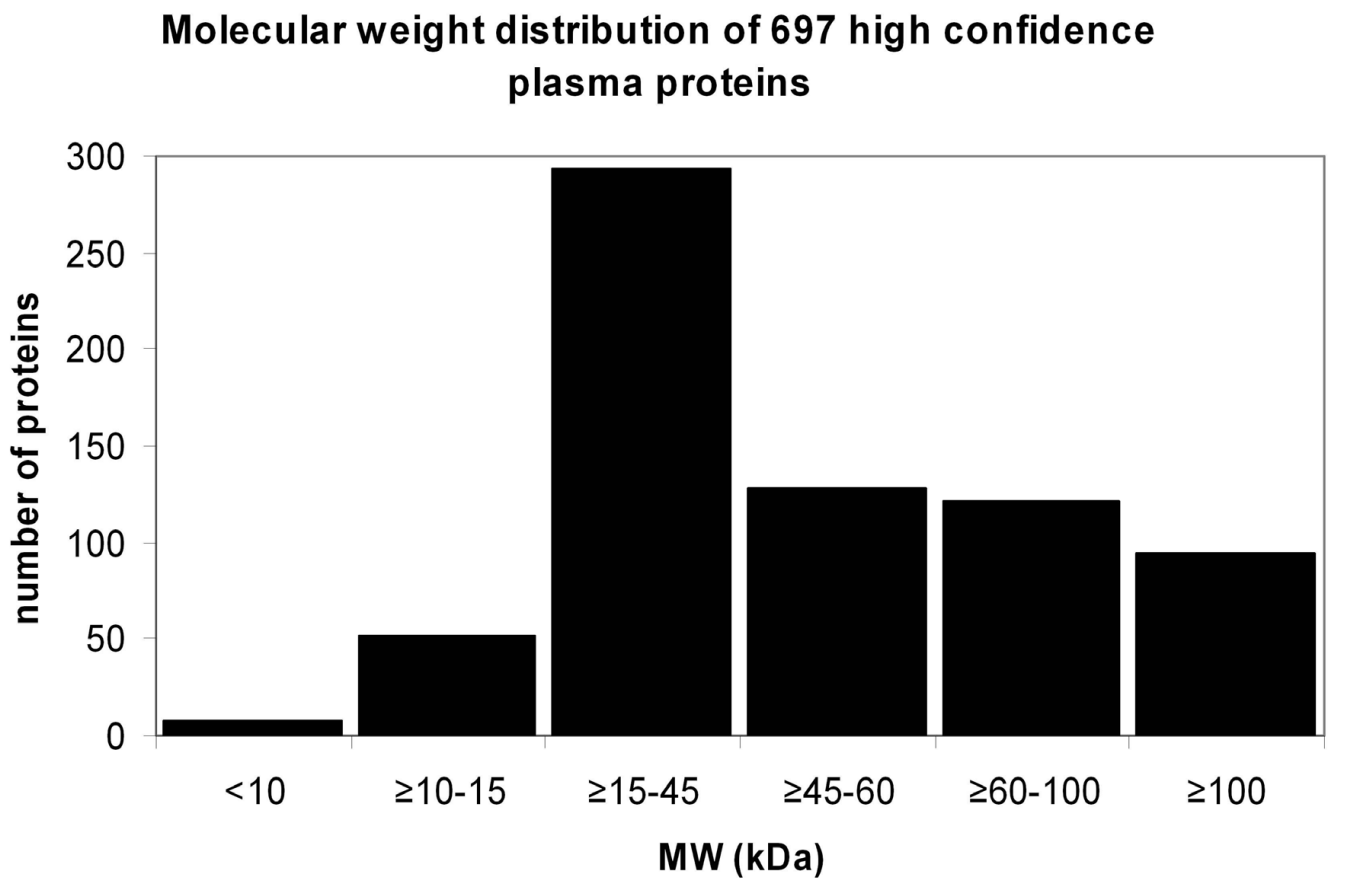
**Histogram showing the molecular weight distribution of the calculated masses of 697 observed plasma proteins**. The calculated protein masses for all proteins from all experiments were categorized into molecular weight groups as indicated so that the number of proteins falling into each molecular weight group is proportional to the height of each bar in the histogram.

In Figure 3 we depicted the number of proteins *versus *their MW. 346 proteins (49.6%) were identified with a MW < 45 kDa, and 351 proteins (50.4%) with a MW ≥ 45 kDa. From those 346 proteins with a MW < 45 kDa, 6 proteins had a MW < 10 kDa, 52 proteins had a MW of 10 ≤ MW < 15 kDa, and 288 proteins fall in the group of 15 ≤ MW < 45 kDa. Furthermore, our analysis shows that 474 proteins of the 697 identified had a MW < 60 kDa. It should be noted that we have not considered the possibility of molecular weight-altering post-translational modifications such as glycosylation due to the lack of comprehensive protein modification data.

Proteins with a MW of <15 kDa are freely filtered in the glomeruli; proteins up to 45 kDa are quite rapidly filtered and proteins between 45 to 60 kDa only restrictedly. Plasma proteins larger than 60 kDa are not filtered through the kidney. It is worthy of note that the MW of approximately half of our identified proteins (346, or 49.6%) allows their unrestricted clearance through kidney filtration due to their MW < 45 kDa. In order to be retained for an extended period in the plasma, these proteins would need to be bound to larger carrier proteins or be subject to some other retention mechanism such as the inclusion of the protein in a complex.

Earlier we mentioned that 49.6% of the 697 identified proteins could be easily filtered through the kidney; thus, the 50.4% remaining proteins could possibly reside for an extended time period in the plasma because they have a MW larger than 45 kDa. However, having a MW > 45 kDa doesn't necessarily make a protein an extracellular protein. Some of the proteins with a MW > 45 kDa will of course be cellular proteins, such as the chaperone-like 'heat shock cognate 71 kDa protein' found in our list, or a bundling protein such as the 'alpha-actinin 1', which is approximately 100 kDa in size. Is there congruence between the number of extracellular proteins predicted by GoMiner (see below) and the number predicted according to MW? There certainly is since most true plasma proteins have a MW above filtration cut off. However, this equation is not as precise, nor as simple as that. Not all of the proteins that are classified as 'extracellular' and which are not part of the 'extracellular matrix' will be plasma proteins. Extracellular proteins such as heparin cofactor II or transforming growth factor-beta induced protein IG-H3 may also be found in the extracellular space (also known as intercellular or interstitial space). Also, some proteins cannot be clearly classified as 'cellular' or 'extracellular', such as uromodulin, a phosphatidylinositol-linked membrane protein, which is also secreted into the urine after cleavage. Another protein, pigment epithelium-derived factor, is found both in retinal pigment epithelial cells and in blood plasma.

**Figure 4:**
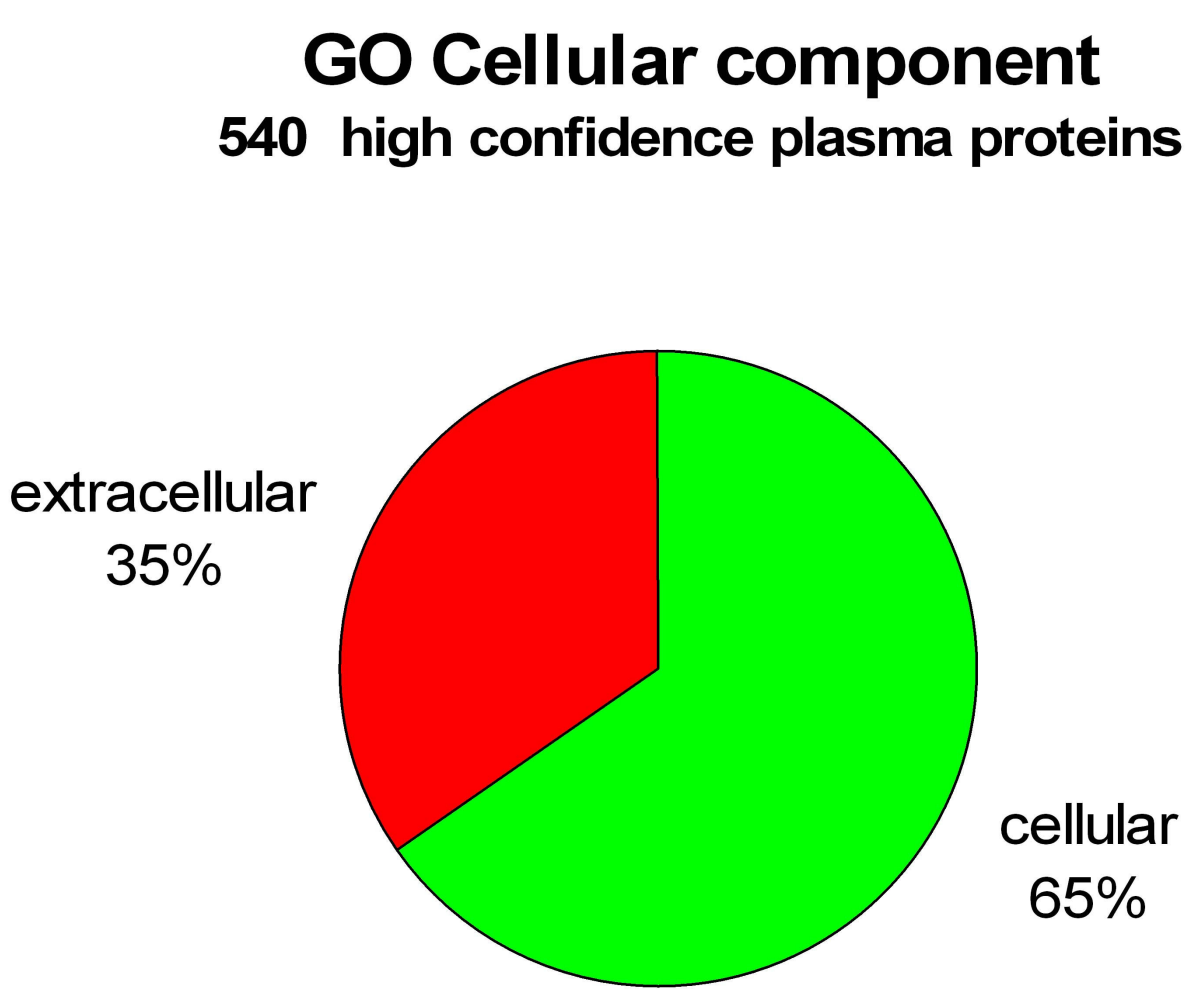
**Pie chart representation of all validated proteins which were categorized as GO cellular component**. Of the 697 plasma proteins identified, 540 of these fell into the 'GO cellular component' category. Of the 540 'GO cellular component' proteins, 392 (65%) were classified as 'cellular' and 208 (35%) were classified as 'extracellular'. 44 of the proteins from the 'extracellular' category were classified as 'extracellular matrix' proteins. Note that because some of the proteins have been reported more than once, the total number of proteins reported for the two categories shown is actually higher than the total number of proteins for the 'GO cellular component'. We have therefore normalized the sum of the cellular and extracellular components to 100%.

According to GoMiner, of the 540 proteins recognized as being part of the cellular component from our list of 697, 208 of the proteins were categorized as 'extracellular' and 392 proteins as 'cellular', although some proteins fall into both categories. Because of this redundancy we have chosen to normalize the sum of the cellular and extracellular categories to 100% for comparison purposes; thus, 35% of the proteins were categorized as extracellular and 65% as cellular (Figure 4). 44 proteins from the extracellular protein group were classified as 'extracellular matrix' proteins, leaving the remaining 164 proteins from the extracellular protein category as possible plasma proteins. As for the remaining 157 proteins not categorized by GoMiner as cellular components, their subsequent classification could potentially alter the present profile.

It is interesting to consider the number of proteins that were found to have a signal peptide, since the presence of a signal peptide indicates that a protein is normally secreted and may thus be a 'true' plasma protein. Our software application that made it feasible to retrieve information based on accession number from the SwissProt/UniProt and NCBI databases for each of our identified proteins also facilitated deposition of signal sequence information into our database. Overall, 44% of our list containing 697 proteins was reported to have a signal sequence, while in 56% of the cases there was no evidence in the literature indicating the presence of a signal sequence (data not shown). In the cases where a signal sequence is not reported, this of course does not exclude the possibility that these proteins have one. As indicated by Swiss-Prot, NCBI and other on line data sources, the presence of a signal sequence was either not investigated or it could not be inferred from similar sequences. For all of the 'hypothetical' proteins, for example, no data are given regarding the presence or absence of a signal sequence.

It seems reasonable to assume that the majority of the 208 proteins (35%) classified as 'extracellular' by GoMiner should have a signal sequence. 90% (188/208) of these extracellular proteins were indeed reported to have a signal sequence. It is unclear why the remaining 10% do not possess one.

The fact that 32% (125/392) of the proteins classified as 'cellular' by GoMiner also have a signal sequence complicates the issue further. Proteins that are localized in the endoplasmic reticulum (ER), for example, are likely to have a signal sequence. This is the case for calreticulin, which is localized in the ER lumen and is also reported to have a signal sequence according to GoMiner and SwissProt. According to Swiss-Prot, Di-N-acetylchitobiase is a lysosomal protein which is involved in the degradation of asparagine-linked glycoproteins, has a signal sequence and is categorized by GoMiner as a cellular-cytoplasm protein.

To cloud the issue further, we mentioned that having a MW > 45 kDa does not necessarily make a protein an extracellular one and thus having a MW of < 45 kDa is no guarantee that a protein is cellular. Bound to larger carrier proteins, proteins of 45 kDa or less may well exert their function as plasma proteins. Complement factor H-related protein 4, which is involved in complement regulation and has a MW of 38.5 kDa, is expressed in the liver and is secreted into the plasma where it was found to be associated with lipoproteins [16] [UniProtKB/Swiss-Prot entry Q92496]. Platelet factor 4 has a mass of ca. 11 kDa and is bound to a proteoglycan molecule that is released during platelet aggregation. It would be interesting to know the correlation between the plasma protein MW and the degree of carrier protein binding, but it is likely that this correlation is sequence dependent and would not be trivial to ascertain.

**Figure 5:**
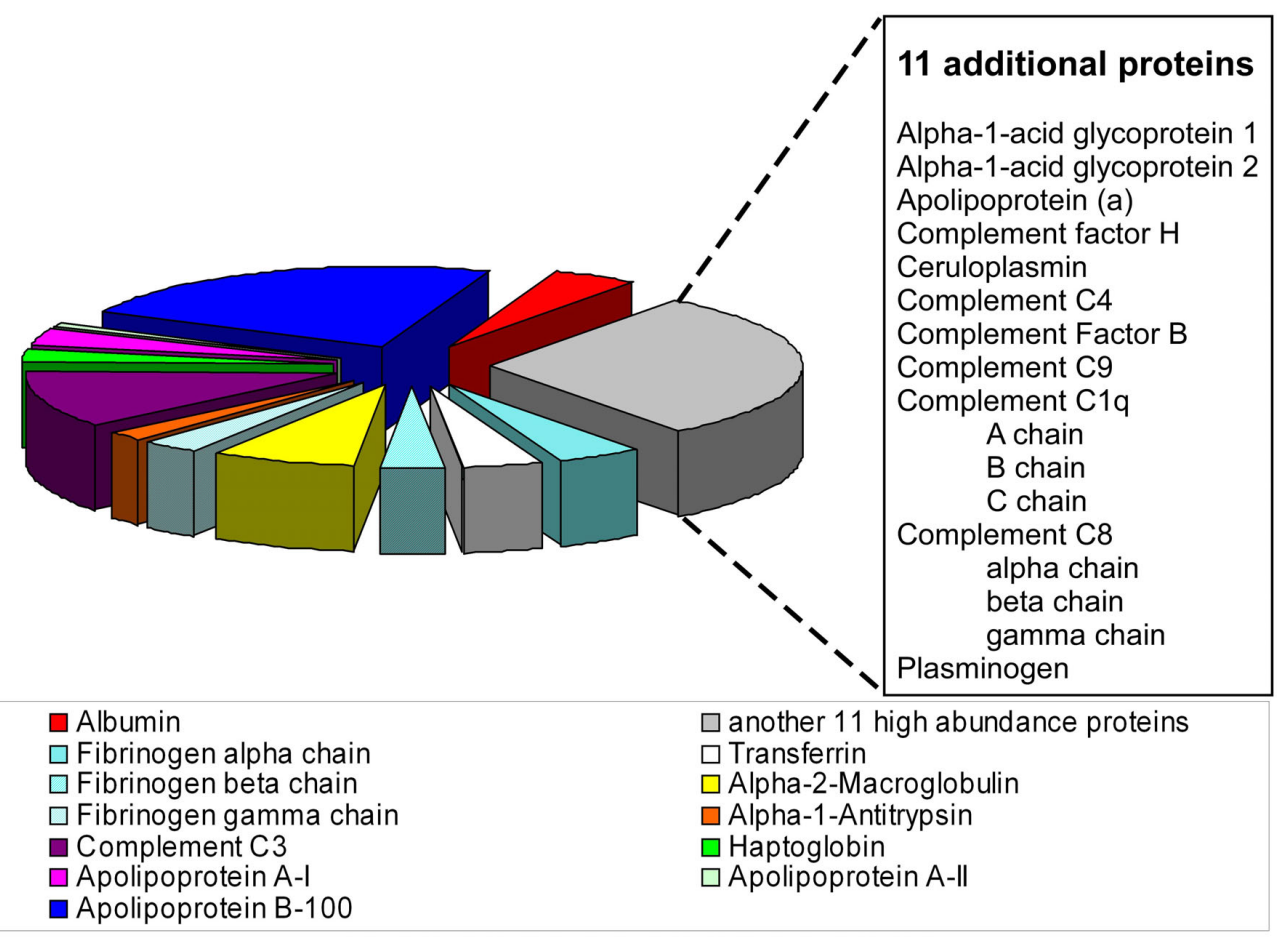
**Pie chart representation of the 21 most highly abundant plasma proteins from our set of 697 proteins across all experiments**. 10 of the 21 most highly abundant plasma proteins from our analysis are depicted in the pie chart. The small table to the right of the pie chart contains the next 11 most abundant proteins. The size of each pie piece is proportional to the number of unique peptides that we sequenced for that particular protein, relative to the total number of peptides for all 21 proteins depicted.

Without a doubt, the biggest challenge in the study of the human plasma proteome is overcoming the detection problems associated with its large protein concentration range, which spans more than 10 orders of magnitude. High abundance proteins mask the low abundance proteins, making the identification of the latter extremely difficult or even impossible, using current technology. However, both high and low abundance proteins can be clinically meaningful and can also be the subjects of clinical assays. Approximately 10 of the most abundant proteins represent roughly 90% of the total protein mass in human plasma, while another 10+ most highly abundant proteins account for an additional 9% of the total protein mass [17]. Hence, slightly more than 20 proteins account for approximately 99% of the total protein mass in plasma. We plotted all of the proteins reported by Schuchard et al. [17] as a function of the number of valid, non-redundant peptides we isolated for each protein (Figure 5). We did not, however, include the immunoglobulins or prealbumin in our analysis. We are aware that counting peptides is not quantitative and that the molecular weight of the proteins has an impact on this type of analysis, but it still provides a crude estimate of the abundance of these proteins. An estimated 10,000 proteins reside in the remaining 1% of the plasma protein mass [18]. Among these are proteins of very low concentration, such as hormones, cytokines and tissue leakage products. For example, two of the proteins we identified, myotrophin and C-reactive protein, are of clinical relevance. Both are novel cardiac biomarkers in heart failure diagnosis. Unlike acute coronary syndromes, the definition of heart failure is a bedside diagnosis based on clinical signs and symptoms rather than any stand-alone test result. The use of biomarkers in the diagnosis and management of heart failure may thus facilitate better clinical judgment. Myotrophin is a small protein of about 13 kDa. It was identified with 4 valid, non-redundant peptides of 10, 13, 17 and 20 amino acids in length. The three peptides having lengths of 10, 13 and 17 residues possess an MS3 spectrum in addition to their MS2 Mascot scores, giving rise to scores of 70, 131 and 176 (summed score of 377), respectively. C-reactive protein (ca. 25 kDa) occurs in plasma at concentrations of 6.8 – 820 × 10^-8 ^g/ml [19] and is several orders of magnitude less in concentration than abundant proteins such as serum albumin, which is found at a concentration of 3.5 – 5.2 × 10^-2 ^g/ml (Table 3). It was identified with 8 valid, non-redundant peptides of 7, 8, 10 (2 × 10 mers), 11 (2 × 12 mers), 12 and 15 amino acids in length, with MS2 or MS3 scores of 135, 39, 123, 104, 115, 35 (no MS3), 116, and 35 (no MS3) respectively, and a summed score of 702. Despite their low abundance and small MW, the correct identifications of both of these proteins were made with high confidence [see Additional file 2].

**Table 3:** Plasma protein concentration ranges of selected proteins.

Plasma protein	Known protein concentrations (g/ml)	Distinct peptides	Mass (Da)
Serum albumin	3.5 – 5.2 × 10^-2^	94	69367
Fibrinogen (alpha chain)	2.0 – 4.0 × 10^-3^	96	94973
Alpha-2-macroglobulin (male adult)	0.9 – 4.0 × 10^-3^	164	163278
Alpha-1-antitrypsin	7.8 – 20 × 10^-4^	44	46737
Haptoglobin	3.0 – 22 × 10^-4^	52	45205
Transthyretin, Thyroxine-binding prealbumin	2.8 – 3.5 × 10^-4^	19	15887
Ceruloplasmin	1.5 – 6.0 × 10^-4^	95	122205
Prothrombin	1.0 × 10^-4^	53	70037
Fletcher factor (Plasma kallikrein precursor)	5.0 × 10^-5^	37	71370
Complement component C6	4.8 – 6.4 × 10^-5^	61	104844
Complement component C9	4.7 – 6.9 × 10^-5^	32	63174
Hageman factor (Coagulation factor XII)	2.9 × 10^-5^	27	67818
Complement C1r component	2.5 – 3.8 × 10^-5^	45	80174
Properdin (Factor P)	2.4 – 3.2 × 10^-5^	2	51276
Complement C2	2.2 – 3.4 × 10^-5^	45	83268
Von Willebrand factor	7 × 10^-6^	55	309299
Stuart factor (Coagulation factor X)	5.0 × 10^-6^	18	54732
Christmas factor (Coagulation factor IX)	4.0 × 10^-6^	16	51748
Transferrin soluble receptor (adult), Serotransferrin	0.8 – 1.8 × 10^-6^	89	77050
Proconvertin (Coagulation factor VII)	1.0 × 10^-6^	*	53043
Mannose-binding protein C (MBP)	0.3 – 4.1 × 10^-6^	14	26144
Beta-2-microglobulin	8.0 – 24 × 10^-7^	8	13715
Antihemophilic factor (Coagulation factor VIII)	1.0 × 10^-7^	---	267009
C-reactive protein, splice isoform 1	6.8 – 820 × 10^-8^	8	25039
Insulin-like growth factor II	9.9 – 50 × 10^-8^	5	20140
Myoglobin	6.0 – 85 × 10^-9^	4	17053
Prolactin (male), awake	1.0 – 7.0 × 10^-9^	---	25876
Insulin	2.0 – 8.4 × 10^-10^	---	11981

Included in the table are proteins identified in our high confidence set of plasma proteins that represent a wide range of protein molecular weights and which have known plasma protein concentrations. Also included are some proteins that are known to be of higher abundance, but which were not detected in our experiments, and these were included to demonstrate that fact. The protein name, known plasma protein concentration, the number of unique peptides identifying the protein, the summed Mascot score and the MW is shown and the table has been sorted in descending order with the protein possessing the highest known protein concentration listed first.

--- indicates that this entry was not identified in this study.

* indicates that this entry was identified but not validated.

The LTQ-FT and oribitrap mass spectrometers that we employed in this study can be considered to be cutting edge technology when it comes to mass spectrometry. However, despite the extremely high dynamic range covered by these instruments, the overall plasma protein concentration range currently resolvable is only up to 7 orders of magnitude (Table 3). We seem to be able to comprehensively cover up to about 4 orders of magnitude. Beyond this the detection of a protein is not guaranteed and indeed, the less abundant a protein is and the smaller its MW, the more unlikely is its detection. However, the unprocessed precursor of anti-hemophilic factor (coagulation factor VIII) was not detected despite its large MW of 267 kDa. At the same time, proteins that are 1 to 2 orders of magnitude less abundant and with much smaller MW, such as C-reactive protein and myotrophin, could be conclusively identified.

As to the content of our high confidence set of 697 proteins, which excludes immunoglobulins, a simple query of the BPPD revealed the identification of 30 proteins that are annotated as 'hypothetical' and 31 that are annotated as 'keratin'. 13 proteins are annotated as 'kinase' and 16 proteins as 'growth factor' [see Additional file 2]. Based on their annotation and/or tissue specificity and subcellular location, 66 proteins are readily identifiable as true 'plasma' proteins (not plasma membrane).

Furthermore, we have provided a short selection of proteins that have important biological function(s) and/or have some role in a disease process (Table 4).

**Table 4:** Selected proteins and their possible involvement in diseases.

Accession	MW	Protein name	Function	Disease
Q15848	26414	Adiponectin	Hematopoiesis, immune system; fat metabolism and insulin sensitivity.	Adiponectin deficiency; obesity insulin resistance, diabetes type 2.
P37840	14460	Alpha-synuclein, isoform 1, 2, or 3	Regulation of dopamine release and transport. Decreased caspase 3 activation.	Defects in SNCA cause autosomal dominant Parkinson disease 1 and Lewy body dementia (DLB).
P02741-1	25039	C-reactive protein, splice isoform 1	Enhances host defense.	Inflammation, heart disease biomarker.
P06703	10180	Calcyclin (Prolactin receptor associated protein)	Preferentially expressed during quiescent fibroblast proliferation.	It is inducible by growth factors and overexpressed in acute myeloid leukemias
P31944	27680	Caspase-14	Apoptosis.	
P07339	44552	Cathepsin D	Acid protease active in intracellular protein breakdown.	Disease pathogenesis: breast cancer, possibly Alzheimer's disease.
P81605	11284	Dermcidin	Neuron survival; phosphatase and antimicrobial activity.	
Q99497	19891	DJ-1 protein (Oncogene DJ1)	Androgen receptor-dependent transcription regulator; prevents aggregation of SNCA; protects neurons from oxidative stress and cell death; role in fertilization.	Early-onset Parkinson disease 7 (PARK7).
P23142-1	77261	Fibulin-1, splice isoform 1 or D	Cell adhesion/migration, organization of ECM, haemostasis and thrombosis, modulation of APP, tumor suppressor.	human breast cancer; synpolydactyly (limb malformation)
P23142-4	74462	Fibulin-1, splice isoform 4 or C		human breast cancer; does not seem to be implicated in synpolydactyly
O75636-1	32903	Ficolin 3, splice isoform 1	Lectin activity.	Systemic lupus erythematosus (SLE).
P16930	46374	Fumarylacetoacetase	Not found	Defects in FAH are the cause of tyrosinemia type I.
Q7M4S4	2046	Granulocyte inhibitory protein	Inhibits the biological activity of polymorphonuclear cells.	
P01344-1	20140	Insulin-like growth factor II, splice isoform 1	Growth-promoting activity; fetal development.	
P05362	57826	Intercellular adhesion molecule-1	ICAM proteins are ligands for the leukocyte adhesion LFA-1 protein (Integrin alpha-L/beta-2).	
P13473	44961	Lysosome-associated membrane glycoprotein 2, splice isoform 1 or 2	Lysosomal maintenance; intracellular signal transduction.	Implicated in tumor cell metastasis.
P02144	17053	Myoglobin	Reserve O_2 _supply, O_2 _movement within muscles.	
P58546	12764	Myotrophin	Cerebellar morphogenesis.	Seems to be associated with cardiac hypertrophy.
P22392	17401	NM23-LV (contains Nucleoside diphosphate kinase B sequence)	Nucleoside diphosphate kinase B is a transcriptional activator of the c-Myc gene; binds DNA nonspecifically.	Reduced amounts of Nucleoside diphosphate kinase B in tumor cells of high metastatic potential.
P15531	17149	Nucleoside diphosphate kinase A	Synthesis of nucleoside triphosphates other than ATP.	Neuroblastoma.
P10720	10845	Platelet factor 4 variant	Inhibitor of angiogenesis, endothelial cell chemotaxis.	
P01133	133946	Pro-epidermal growth factor	Growth of epidermal and epithelial tissues.	
P27918	51276	Properdin (Factor P)	Alternate complement pathway; binds C3- and C5- convertase enzyme complexes.	Properdin deficiency (PFD); higher susceptibility to bacterial infections; especially meningococcal.
P61019	23546	Ras-related protein Rab-2A	Protein transport; endoplasmic reticulum to Golgi complex.	
P51149	23490	Ras-related protein Rab-7	Protein transport. Vesicular traffic.	Charcot-Marie-Tooth disease type 2B (CMT2B).
Q12913	145927	Receptor-type tyrosine-protein phosphatase eta	Mechanism of contact inhibition of cell growth.	Cancers of colon, lung, and breast.
P48594	44854	Squamous cell carcinoma antigen 2	Protease inhibitor; host immune response modulator.	Seems to also be secreted in plasma by cancerous cells but at a low level.
Q15582	74681	Transforming growth factor-beta induced protein IG-H3	Cell-collagen interactions; endochondral bone formation.	Corneal dystrophy Groenouw type I (CDGG1).
P07911	69761	Uromodulin	Not known. Possible regulation of cytokines.	Familial juvenile hyperuricemic nephropathy (HNFJ); medullary cystic kidney disease 2 (MCKD2).

We have provided a short selection of proteins that we identified in our experiments and that have important biological functions and/or may have some role in a disease process. The table includes a description of the protein function, the disease involved, the primary accession number of the protein, and the molecular weight and name of the protein and has been arranged alphabetically according to protein name.

### Comparison of individual experiments

**Figure 6:**
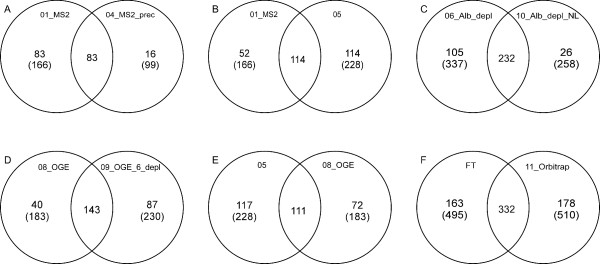
**Venn diagram representations of comparisons between pairs of individual plasma experiments**. Individual experiments are represented by circles, with compared experiment pairs being depicted by overlapping sets of two circles. The number of proteins identified in both members of a compared set of experiments is given in the intersection region of the circles. The number of proteins that are unique to an individual experiment is shown outside of the intersection region, along with the total number of proteins identified for that individual experiment, shown in parentheses. The plasma experiment represented by each circle is represented at the top of the respective circle, outside of the intersection region (panel A, for example, shows a comparison of experiments 01_MS2, designated "01'', and 04_MS2_prec, designated "04''). The experimental conditions used in each experiment can be found in Table 1. Panel F shows a comparison between all 7 experiments performed on the FT and the last experiment which was performed on the Orbitrap.

**Figure 7:**
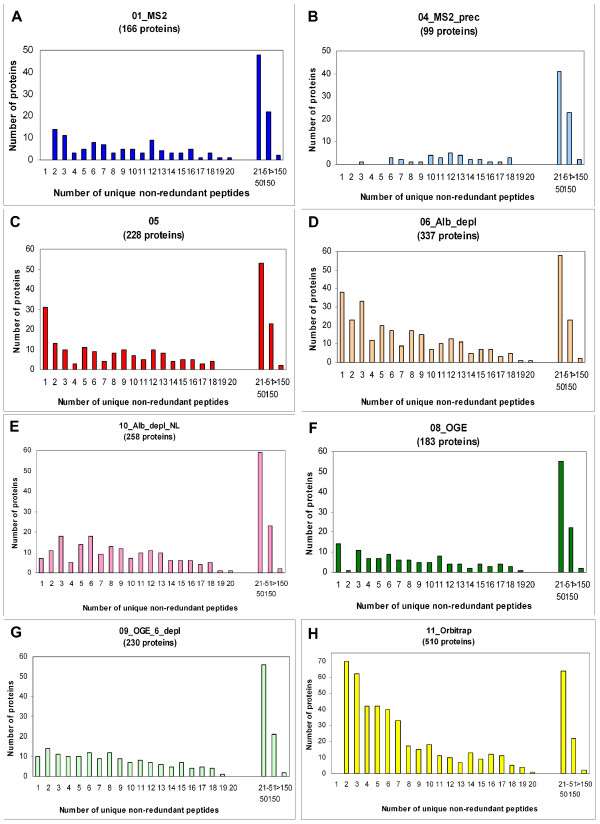
**Histograms showing the number of proteins identified with a given number of unique peptides for each individual experiment**. The number of validated, non-redundant peptides was calculated for each protein identified within the context of each individual experiment, and proteins having identical numbers of peptides were grouped together and plotted as indicated. The Y-axes (number of proteins) for the experiments indicated in panels C and D and in A, B, E, F and G have been standardized in order to facilitate cross-experiment comparison.

**Figure 8:**
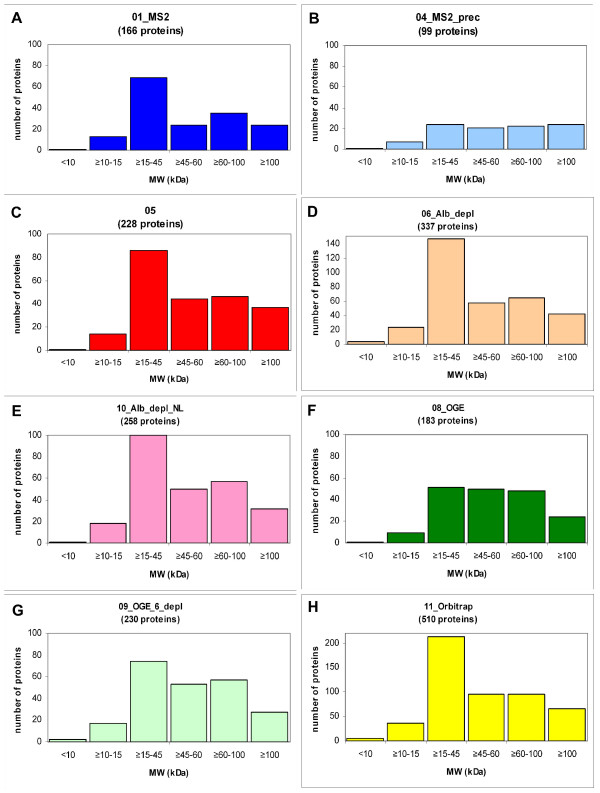
**Histograms depicting the molecular weight distribution of the identified proteins in each of the 8 experiments**. The proteins from each individual experiment were grouped according to their calculated molecular weight and plotted as indicated. All Y-axis scales except for the ones in panels D and H have been standardized to facilitate cross-experiment comparison.

All of the protein validation rules that we applied to the combined set of 697 proteins were also applied to each individual experimental dataset before comparison. In the course of analysing the data from the different experimental treatments listed in Table 1, we noted some interesting observations. In performing the comparisons, we looked at the total number of proteins identified and those found to be in common between experiment pairs (Figure 6). Furthermore, the valid, non-redundant peptide distribution vs. proteins identified (Figure 7), as well as the MW distributions (Figure 8) for the different experiments were investigated.

In order to determine whether choosing restricted precursor selection would affect our identification of proteins from samples, we compared the results of 01_MS2 and 04_MS2_prec (Figure 6, panel A). Interestingly, there were only 16 proteins that were identified in 04_MS2_prec that were not identified in 01_MS2. Even though more plasma sample was applied to the 1D PAGE gel in experiment 04_MS2_prec, significantly more proteins were identified in 01_MS2. Additionally, 04_MS2_prec was the experimental protocol that identified the least number of proteins of any of the experiments, suggesting that several hundred more μg of protein could not make up for the seemingly disadvantageous MS setting of restricted precursor selection chosen for 04_MS2_prec. Note that single peptide identifications do not appear in 01_MS2 and 04_MS2_prec because our validation criteria demanded an MS3 spectrum, which is precluded in these experiments which were only MS2-based (Figure 7, **panels A and B**). We also plotted the molecular weight distribution of the proteins identified in 01_MS2 (Figure 8, **panel A**) and 04_MS2_prec (Figure 8, **panel B**) and found that many of the additional proteins identified in 01_MS2 fell into the 15–45 kDa range.

In order to determine the effect of the amount of plasma protein loaded on the gel with respect to the number of proteins identified, we performed a comparison between 01_MS2 and 05 (Figure 6, **panel B**). We identified 166 proteins in 01_MS2 and 228 proteins in 05 but because there are two variables involved in this comparison (namely protein loading and collection of MS3 data), we eliminated single peptide identifications from experiment 05 in order to negate the MS3 variable. This left us with 197 proteins identified in 05 (without MS3) and 166 proteins identified in 01_MS2. Since we loaded about 300 ug more protein (67%) in 05 compared to 01_MS2, the identification of more proteins in 05 doesn't seem surprising. However, it should be noted that the extra protein loaded in 05 resulted in only 19% more proteins identified compared to experiment 01_MS2.

The utilization of more applied protein and MS3 in 05 *versus *01_MS2 did not have an appreciable impact on the profile depicting unique peptide distribution (Figure 7, **panels A and C**) except for the single peptide peak due to MS3. The MW distributions of the identified proteins in 05 and 01_MS2 (Figure 8, **panels C and A **respectively) appear highly similar and are unaffected by the removal of proteins identified with a single peptide since the additional proteins identified in 05 are found to be equally distributed across all MW ranges.

In order to examine the effect of protease inhibitor addition to plasma samples, we compared experiment 06_Alb_depl with 10_Alb_depl_NL where we obtained 337 vs 258 proteins identified respectively (Figure 6, **panel C**). Because the MS3 settings for these experiments were different (neutral loss-dependent MS3 was used in this study in experiment 10_Alb_depl_NL only) we negated the MS3 results by removing single peptide-based protein identifications from both experiments, thus allowing a comparison based only on the addition of protease inhibitors. Removal of single peptide identifications left us with 299 proteins identified in 06_Alb_depl and 251 proteins identified in 10_Alb_depl_NL. In our hands, the addition of protease inhibitors (10_Alb_depl_NL) did not result in identifying more proteins, but rather we obtained 48 fewer proteins than in 06_Alb_depl.

Neutral loss-dependent MS3 detects the loss of phosphoric acid (ca. 98 Da) from the precursor ion in a MS2 scan and initiates MS3 fragmentation analysis of the neutral loss precursor ion. As expected, the use of this method reduced the number of proteins with 1 peptide (Figure 7, **panels D and E**), which is also reflected in the lower number of proteins with smaller MW (< 45 kDa) (Figure 8, **panels D and E**). Although the neutral loss-dependent MS3 setting used in experiment 10_Alb_depl_NL was employed to detect phosphopeptides, the results obtained were unclear and therefore are not reported here.

To determine the effect of protein depletion on the number of proteins identified, a somewhat different approach was used, and different results were seen for 08_OGE and 09_OGE_6_depl (Figure 6, **panel D**). Both experiments employed OGE separation, while 09_OGE_6_depl was additionally depleted of six high abundance proteins, including albumin, transferrin, haptoglobin, alpha-1-antitrypsin, IgA and IgG. Since the immuno-depletion column employed removes from approximately 65% to 85% of the total plasma protein (Agilent, personal communication; [20]), the amount of protein loaded in the 09_OGE_6_depl_sample was correspondingly reduced to 650 ug to compensate for the loading of the non-depleted 1800 ug from 08_OGE. We found that experiment 09_OGE_6_depl produced more validated proteins than 08_OGE (Figure 6, **panel D**), suggesting that the depletion step may have been somewhat advantageous. The majority of the additional 47 proteins in 09_OGE_6_depl as compared to 08_OGE were identified with up to 10 peptides (Figure 7, panels G and F respectively), and can be found mostly in the lower MW range of up to 45 kDa (Figure 8, panels G and F respectively), supporting the idea that depletion may facilitate the identification of otherwise 'overshadowed' smaller proteins.

In order to compare the 1D-PAGE and OGE separation methods, we performed two experiments, 05 and 08_OGE, neither of which employed a depletion step. Even though 2.4 times more protein was loaded in 08_OGE, more proteins were identified in the 05 experiment (Figure 6, **panel E**). This suggested that in our hands the 1D-PAGE separation seemed to be more effective than the OGE technique.

Experiment 05 (Figure 7, **panel C**) identified twice as many proteins with 1 peptide and more proteins with 2 peptides than 08_OGE (Figure 7, **panel F**). With respect to the MW distribution, experiment 05 had 1.7 times more proteins with a MW of up to 45 kDa than 08_OGE (Figure 8, **panels C and F**). These observations support the notion that plasma separation by OGE rather than by 1D PAGE may thus adversely affect the identification of proteins of low abundance and/or in the small MW range.

Finally, a comparison between the data obtained from all 7 plasma gels analysed on the FT with the results from experiment 11_Orbitrap (Table 1), which was analysed via the Orbitrap confirm that the Orbitrap is a high performance mass spectrometer. Even though significantly less plasma sample was applied to the Orbitrap compared with the 7 samples measured on the FT, the results of both analyses are comparable. Both instruments yielded about 500 high confidence proteins and 332 (ca. 65%) identical protein identifications (Figure 6, panel F). Also, as seen on Figure 7, panel H, none of the 510 proteins identified on the Orbitrap was validated with only one peptide (MS3 was not performed), whereas 93 proteins from the 495 identified collectively by all FT runs were validated with 1 peptide using MS3. Figure 8, panel H shows that the MW distribution for the Orbitrap data is quite similar to the data obtained in several of the FT experiments, notably 06_Alb_depl (Figure 8, panel D) and 10_Alb_depl_NL (Figure 8, panel E). The serum samples were depleted prior to MS analysis in all three experiments.

**Figure 9:**
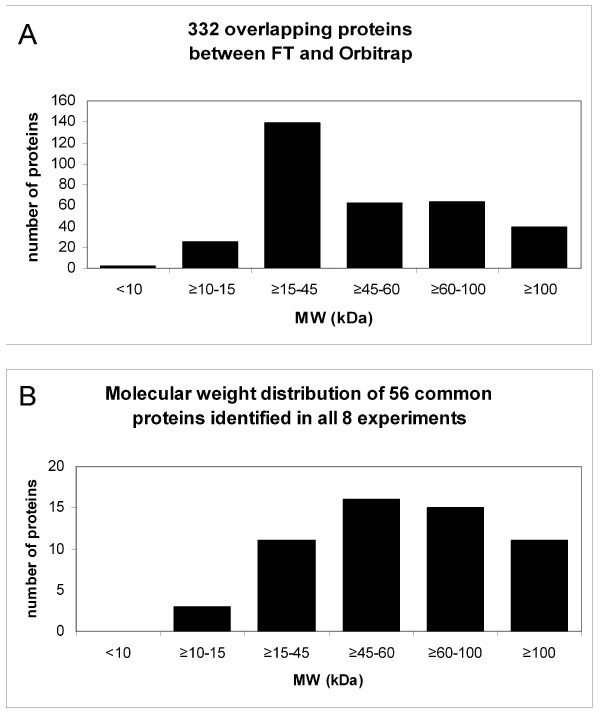
**Histograms showing the molecular weight distribution of shared proteins**. The calculated molecular weights of the proteins determined to be in common between each set of indicated experiments were categorized and plotted. Panel A depicts a comparison of the proteins found to be in common between the FT (seven independent experiments) and the Orbitrap. Panel B shows the calculated molecular weights from the 56 proteins identified in all experimental approaches.

In comparing individual experimental results, the expectation is that proteins of higher molecular weight and/or higher abundance should have a higher likelihood of being identified across experiments due to the associated higher probability of isolating and identifying peptides from those two groups of proteins. Although we did not have comprehensive data available to us concerning protein abundance, we thought it would still be interesting to know whether the common proteins identified in a given set of experiments favor a certain MW range. Figure 9, **panel A **illustrates the MW distribution of the 332 proteins that were identified by both the FT and the Orbitrap. This histogram profile is very similar to the one depicting the MW distribution of all 510 proteins isolated using the Orbitrap (Figure 8, **panel H**) and also to the profile generated by plotting the MW data from all seven FT-based experiments (not shown). Since we used what we considered to be the best plasma handling method (depletion/1D-PAGE) for the Orbitrap experiment, it came to us as no surprise that the MW profile of the shared 332 proteins isolated using the FT and Orbitrap should produce similar MW profiles. In contrast to this, a comparison of all individual experiments to each other produced a set of only 56 proteins identified across all experiments. In this case the MW profile of the proteins found across all experiments shows a definite bias toward proteins of higher molecular weight, as expected (Figure 9, **panel B**). Almost all of these 56 proteins are classical plasma proteins, exerting their function within the plasma (Table 5). The low numbers of shared proteins between all experiments highlights the high variability in protein identification seen when using different methods and instruments in MS-based proteomic analysis.

**Table 5:** List of the 56 proteins found in all 8 experiments.

Primary accession number	IPI number (version 3.25)	Protein MW	Protein name
P43652	IPI00019943	69069	Afamin [Precursor]
P02763	IPI00022429	23512	Alpha-1-acid glycoprotein 1 precursor
P19652	IPI00020091	23603	Alpha-1-acid glycoprotein 2 [Precursor]
P01011	IPI00431656/IPI00550991/IPI00411920	47651	Alpha-1-antichymotrypsin [Precursor]
P04217	IPI00022895/IPI00644018	54273	Alpha-1B-glycoprotein [Precursor]
P02765	IPI00022431	39325	Alpha-2-HS-glycoprotein [Precursor]
P01023	IPI00478003	163278	Alpha-2-macroglobulin [Precursor]
P02760	IPI00022426	39000	AMBP protein [Precursor]
P01008	IPI00032179	52603	Antithrombin-III [Precursor]
P02647	IPI00021841	30778	Apolipoprotein A-I [Precursor]
P02652	IPI00021854/IPI00382587	11175	Apolipoprotein A-II [Precursor]
ENSP00000350425	IPI00304273	45399	Apolipoprotein A-IV [Precursor] (Apo-AIV)
P04114	IPI00022229	515563	Apolipoprotein B-100 [Precursor]
P02655	IPI00021856	11284	Apolipoprotein C-II precursor
P02656	IPI00021857/IPI00657670	10852	Apolipoprotein C-III [Precursor]
P05090	IPI00006662	21276	Apolipoprotein D [Precursor]
P02649	IPI00021842	36154	Apolipoprotein E [Precursor]
O14791-2	IPI00514475/IPI00186903	45871	Apolipoprotein-L1 [Precursor], splice isoform 2
P04003	IPI00021727	67033	C4b-binding protein alpha chain [Precursor]
P00450	IPI00017601	122205	Ceruloplasmin [Precursor]
P10909	IPI00291262	52495	Clusterin [Precursor]
P00748	IPI00019581	67818	Coagulation factor XII [Precursor] (Hageman factor)
P02747	IPI00022394	25774	Complement C1q subcomponent, C chain [Precursor]
P09871	IPI00017696	76685	Complement C1s subcomponent [Precursor]
P06681	IPI00303963	83268	Complement C2 [Precursor]
P01031	IPI00032291/IPI00169407	188331	Complement C5 [Precursor]
P13671	IPI00009920	104844	Complement component C6 [Precursor]
P00751-1	IPI00019591	85533	Complement factor B [Precursor], Splice isoform 1
P08603-1	IPI00029739	139071	Complement factor H [Precursor], splice isoform 1
P02671-1	IPI00021885	94973	Fibrinogen alpha chain [Precursor] (Fibrinogen alpha/alpha-E chain precursor), Splice isoform Alpha-E
P02675	IPI00298497	55928	Fibrinogen beta chain [Precursor]
P02679-2	IPI00021891/IPI00167009/IPI00219713	49496	Fibrinogen gamma chain [Precursor], splice isoform 2 (isoform Gamma-A)
P06396	IPI00026314/IPI00377087	85698	Gelsolin [Precursor], plasma (Actin-depolymerizing factor)
P00738	IPI00478493/IPI00641737/IPI00431645	45205	Haptoglobin [Precursor]
P02790	IPI00022488	51676	Hemopexin [Precursor]
P05546	IPI00292950	57071	Heparin cofactor II [Precursor]
P04196	IPI00022371	59579	Histidine-rich glycoprotein [Precursor]
Q3B7H5	IPI00028413	99849	Inter-alpha (Globulin) inhibitor H3 – Homo sapiens (Human).
P19827	IPI00292530/IPI00383338	101389	Inter-alpha-trypsin inhibitor heavy chain H1 [Precursor] (ITI heavy chain H1) (Inter-alpha-inhibitor heavy chain 1) (Inter-alpha-trypsin inhibitor complex component III) (Serum-derived hyaluronan-associated protein) (SHAP)
P19823	IPI00305461/IPI00289083	106436	Inter-alpha-trypsin inhibitor heavy chain H2 [Precursor]
NP_000412	IPI00009865	58827	keratin 10 [Homo sapiens]
NP_000217	IPI00019359	62064	Keratin, type I cytoskeletal 9 (Keratin-9)
NP_006112	IPI00220327	66067	Keratin, type II cytoskeletal 1 (Keratin-1, Cytokeratin 1; hair alpha protein)
P03952	IPI00654888	71370	Plasma kallikrein [Precursor] (Fletcher factor)
P05155	IPI00291866	55154	Plasma protease C1 inhibitor precursor
P02753	IPI00022420	23010	Plasma retinol-binding protein [Precursor]
P00747	IPI00019580	90569	Plasminogen [Precursor]
P00734	IPI00019568/IPI00006618	70037	Prothrombin [Precursor] (EC 3.4.21.5)
P02768	IPI00022434/IPI00745872	69367	Serum albumin [Precursor], splice isoform 1
P02743	IPI00022391	25387	Serum amyloid P-component [Precursor]
Q9Y490	IPI00298994	269767	Talin-1
P07996	IPI00296099	129413	Thrombospondin-1 [Precursor]
P02766	IPI00022432	15887	Transthyretin [Precursor]
ENSP00000273951	IPI00555812/IPI00742696	52918	Vitamin D-binding protein precursor
P04004	IPI00298971	54306	Vitronectin [Precursor]
P04275	IPI00023014	309299	Von Willebrand factor [Precursor]

The table provides the primary accession number, IPI number(s), protein annotation and the molecular weight of all 56 proteins that we found to be in common among our eight different experiments and is sorted alphabetically according to protein name.

Despite everything, many proteins (Table 4) would not have been identified if not for the use of plasma depletion techniques. For example, the smallest protein identified 'Granulocyte inhibitory protein' (2045 Da), was identified in 06_Alb_depl, and another protein of 11 kDa (dermcidin) was identified in 09_OGE_6_depl and 11_Orbitrap only. In fact, myotrophin (13 kDa) was identified in all four experiments where we employed depletion (06_Alb_depl, 09_OGE_6_depl, 10_Alb_depl_NL and 11_Orbitrap), but in no other experiment. In summary, it seems reasonable to recommend plasma depletion as a method to facilitate the identification of lower MW and lower abundance proteins. When the seven experiments measured on the FT are compared, the highest proportion of proteins listed in additional file 2 [see Additional file 2] as well as in Table 4 originated from experiment 06_Alb_depl. When we employed an instrument having a higher sensitivity (Orbitrap), even though much less blood plasma sample was analysed, more proteins were identified. In general, the 1D-PAGE method in combination with serum depletion and if possible, analysis on a high sensitivity instrument such as the Orbitrap, provided the most comprehensive analysis in our hands.

## Discussion

Here we discuss our findings in comparison to the results from HUPO PPP, Anderson *et al *and others.

### Statistics

We identified 697 proteins with very high confidence, 70 proteins of those were identified with only 1 peptide (10%) and 627 proteins with 2 or more peptides (90%). 84 proteins (12%) were identified with exactly 2 peptides, 84 (12%) with exactly 3, 51 (7%) with exactly 4, and 408 proteins (59%) with more than 4 peptides. The combined effort of 35 laboratories, a Bioinformatics group devoted to the PPP, and a rigorous statistical approach, combined with multiple hypothesis testing, led to a reduced set of 889 proteins. For comparison purposes, we removed all proteins that were annotated as immunoglobulins from the HUPO list (94) to obtain a list of 795 remaining high confidence proteins. Of these 795 proteins that were classified as 'high confidence HUPO proteins', 241 were identified with only 1 peptide (30%) and thus 554 with 2 or more peptides (70%). 131 (16%) were identified with exactly 2 peptides, 77 (10%) with exactly 3 peptides and 46 (6%) with exactly 4 peptides. 300 proteins (38%) were identified with more than 4 peptides.

The HUPO endeavor yielded 795 proteins, while our efforts produced 697 proteins, both lists excluding immunoglobulins and rated as 'high confidence'. We believe that the protein/peptide relation of our dataset is quite good, with 697 proteins identified by 10,145 distinct, valid peptides, which calculates to approximately 14.6 peptides per protein on average (14.56) with a median of 6 peptides per protein identified and an overall confidence level of better than 99%. The HUPO high confidence set has a protein/peptide relation with 8942 peptides for 795 proteins, which is equivalent to 11 peptides per protein on average (11.25), with a minimum of 95% confidence. Chan et al. [21] report the identification of 1444 proteins with 2646 peptides. This calculates to only 1.83 peptides per protein on average and accordingly, their overall confidence level was found to be just 90%. It therefore follows that low protein/peptide relations considerably impair the confidence in a dataset.

### Data validation and confidence

Adamski et al. [22] employed a Poisson model to assess a confidence value for protein identifications that they then applied to the HUPO PPP data set. They suggest that of the proteins identified with one peptide, at least 70% are false positives, while 15–30% of proteins identified with exactly two peptides are likely to be false positives. Only about 2–5% of the proteins identified with exactly three peptides are likely to be false. This very high false positive rate is acknowledged by Omenn et al. to be a major problem, especially for identifications based on 1 peptide [5].

Our validation criteria were extremely stringent and only peptides that passed all criteria were included in the protein identification process. In fact, to the best of our knowledge, our set of blood plasma proteins was generated according to the most stringent validation and redundancy criteria published so far. Because the accuracy of protein identification increases with peptide length, we excluded, for example, all peptides that did not have the minimum required length of 7 amino acids, while Adamski et al. eliminated all peptides shorter than 6 amino acids from further analysis [17]. We were also aware of the high risk of incorrect protein identification based on 1 peptide. We therefore discarded all protein identifications based on 1 peptide unless a given peptide was validated by a MS3 spectrum, significantly increasing confidence in peptide identification [7]. Additionally, it was mandatory that the MS3 score have a confidence level of 99.9%. Accordingly, we discarded all identifications based on 1 peptide in assays 01_MS2, 04_MS2_prec and 11_Orbitrap since we did not employ any MS3 in these three experiments. Probabilities of 99% and 95% for the first and second peptide, respectively, were used for protein identification with two peptides, and a minimum of 99% probability for one of three peptides was used for identifications with three peptides. Our decoy database analysis revealed a very low false peptide identification rate of only 0.29 percent across all of our experimental data, reinforcing the notion that the use of such stringent validation criteria produces high confidence data.

Compared with the PPP dataset generated by the collaborative effort of 35 laboratories, our list of identified proteins was not expected to be as extensive. However, because our data were acquired using only two instruments and a single search algorithm (Mascot), and because each peptide was validated identically using the highly stringent criteria, our data were validated homogeneously across all experiments performed. Thus, the huge task of heterogeneous data integration performed by the HUPO Bioinformatics hub was not the key challenge in our case. Yet, the exceptionally careful removal of redundancy which we performed was an incredibly time consuming undertaking. We feel that the strength of our dataset lies in the superior performance of our mass spectrometers and their high mass accuracy, the stringency of our validation criteria, and the homogenous manner in which our data were collected and validated. Validation was performed manually and was later corroborated with the aid of a software script and by database querying. Importantly, data validation was carried out with meticulous accuracy.

The PPP dataset was collected using low and high performance mass spectrometers including LCQs, LTQs, QTOFS, QSTARS and also an FT-ICR by one laboratory, and was processed using different searching algorithms such as Mascot and Sequest. While each mass spectrometer and search engine has its strengths and thus the use of these various technologies could be considered an advantage in the identification process of a diverse proteome such as the plasma proteome, the use of multiple technologies also introduces variables which must be carefully controlled if the data are to be meaningfully compared and/or combined. In our opinion, it is critical that the peptide validation criteria employed for the evaluation of different datasets be homogeneous. Although some integration features were applied to the PPP dataset, no equivalency rules were applied across the search algorithms or for the various 'high confidence' cutpoints [5]. Accordingly, peptide identification was very subjective and peptides of low confidence with lower probability scores were included in protein identifications. This heterogeneity has likely contributed to the high false positive rate of protein identification as described by Adamski, especially for protein identifications based on one peptide.

### Data comparison

In the course of comparing our data with the HUPO data we observed that even though all HUPO proteins have IPI numbers from version 2.21, the high confidence set of 795 proteins (without immunoglobulins) has 319 entries that are missing cross reference information to other databases such as SwissProt/UniProt, making a comparison using this information difficult. Since the IPI datasets change occasionally, we decided to convert all of the HUPO data as well as our data to version 3.25 of the IPI database in order to facilitate a comparison.

A comparison using the converted IPI numbers revealed that 242 of our 697 high confidence proteins matched the HUPO high confidence list, excluding immunoglobulins. However, several circumstances make it likely that this number is not 100% precise. First, we discovered examples of annotations in the HUPO list that are not completely accurate, as well as examples of redundant entries (see below). Second, we verified that our peptides matched the protein sequences referenced by the primary accession number (SwissProt, RefSeq, Ensemble or H-INV), but we did not do this for the IPI numbers that were assigned by Mascot, so we cannot assure a 100% correct assignment of our proteins and peptides to the indicated IPI numbers. However, the 242 matching entries gave us a reasonable measure of the extent of matching proteins from the two datasets without claiming a 100% accurate comparison.

### Redundancy

In the course of our analysis of the HUPO high confidence set we encountered some protein identifications having identical SwissProt and/or IPI accession numbers that appeared to be duplicates. For example, we found that the SwissProt accession numbers P01024, P22792, and Q8N355 each occur twice, while 13 different IPI numbers each appeared twice (data not shown). Visual inspection of the annotations for these entries indicated to us that it may have been appropriate to merge many of the duplicate entries.

There were other entries in the HUPO list that seemed to be redundant even though they had distinct IPI numbers and distinct annotations. In the HUPO list, IPI00293665 (P04259) is annotated as "Keratin type II cytoskeletal 6B", while IPI00296350 (P48669) is annotated as "Keratin type II cytoskeletal 6F". Upon further investigation, we found that according to the EBI, both IPI00293665 and IPI00296350 refer to "Keratin type II cytoskeletal 6B".

In another example, the 7 distinct peptides listed in HUPO's PPP for IPI00293057 (HUPO annotation "carboxypeptidase b-like protein") are also found in the sequence IPI00329775 (HUPO annotation "carboxypeptidase b2"). All 13 of the distinct HUPO peptides listed for IPI00329775 map to the sequence for IPI00329775, but 4/13 peptides do not map to the IPI00293057 sequence. Our observations indicate that these entries should be collapsed into one entry, IPI00329775, since there are no peptides that unambiguously identify IPI00293057.

Care must also be taken in correctly annotating isoforms. For example, the HUPO set identifies ADP-ribosylation factor 3 (IPI00215917). However, the single peptide used to identify this protein also maps to ADP-ribosylation factor 1 (IPI00215914) and the correct annotation should therefore be ADP-ribosylation factor 1 or 3. In a last example, HUPO lists 76 distinct peptides identifying alpha-1-antichymotrypsin isoform 2 (IPI00032215). Upon closer inspection, 35 of the 76 peptides did not match isoform 2, but all 76 of the peptides were found to match alpha-1-antichymotrypsin isoform 1 instead (IPI00550991).

The examples of inaccuracy and redundancy that we identified within the HUPO high confidence list (above) make it evident that much effort is needed to ensure the accuracy of a data set such as we present here. Given that the public databases are in a constant state of flux, disparities in the data, such as those we have listed above, could at least partially be the result of changes to records in the parent databases over time. Unfortunately, search engines like Mascot, Sequest, PepSea or Sonar are not currently 'sophisticated' enough to negate the need for manual confirmation on their proteomic data identification capabilities. Clearly, repetition of scientific experiments using different samples is a basic and vital scientific premise to corroborate data, since the repeated identification of a false positive is less likely as the number of repetitions increases. Even though the massive amount of data obtained from a single experiment already prohibits the feasibility of manual data checking, we still found it necessary to perform such checks, at least for mass spectrometry based proteomics experiments where the goal of the study is to identify a reference data set as a foundation for future work, such as biomarker discovery.

### Data congruence

Anderson et al. [23] investigated the congruence between plasma proteins derived from four different sources. One source was the literature, and the three others were experimental, all MS-based and with immunoglobulin removal, while the separation method was either at the protein level using 2D-gel electrophoresis, at the peptide level using 2D LC of trypsin-digested plasma, or a 'hybrid' experiment with molecular mass fractionation followed by 2D LC. A total of 1,175 proteins were identified from the combined sources and of these, 980 occur in only 1 source. 195 proteins were found in at least 2 of the 4 datasets, 102 proteins were identified in only 2, and 47 proteins were identified in 3 datasets. 46 proteins were found in all 4 datasets, with only one protein having a single transmembrane domain (inter-alpha-trypsin inhibitor heavy chain H1), and one protein without a signal peptide (hemoglobin beta chain). The authors state that the absence of a transmembrane domain and the presence of a signal peptide are characteristics of major plasma proteins. Our set of 697 plasma proteins also identifies 45 proteins of the 46 found in those 4 sources (one entry is an immunoglobulin). In 5 out of the 45 cases the primary accession numbers chosen by Anderson and by us differ, but the proteins identified were identical.

Additionally, Anderson et al. described 47 proteins that were identified in 3 out of 4 sources, 1 of which was an immunoglobulin, leaving 46 non-immunoglobulin proteins. We identified 43 of those 46 proteins as well. The three proteins we did not identify were 60-kDa heat shock protein (we identified heat shock cognate 71 kDa protein and heat shock 90 kDa protein 1, alpha, identified but eliminated with validation), glutamate carboxypeptidase II (84.3 kDa), and phosphoglycerate mutase 2 (28.6 kDa; identified but eliminated with validation, however, we identified phosphoglycerate mutase 1). Due to the technology applied it is reasonable to expect a certain degree of variation in the protein profile identified between MS runs. The molecular masses of the proteins are in the mid- to higher mass category and so were likely not a major deciding factor in determining why we did not identify these proteins.

We did not extensively compare the 102 proteins that Anderson reported to be identified in 2/4 sources because we could not locate this list online. However, we were able to identify some proteins from this category based on visual inspection of the list published in Anderson et al. Adiponectin, cathepsin D, selenoprotein P and squamous cell carcinoma antigen 2 (Anderson reported the antigen 1 variant) are examples of proteins found to be in common between the two studies.

The types of keratins we identified represent what is commonly considered to be due to contamination. It is interesting that the PPP high confidence set of 795 proteins contains 24 (3.0%) 'keratin' entries. Our set of 697 proteins contains 31 (4.4%) keratin entries. It appears as if the keratin contamination problem is universal and is rather equally represented across different research groups. However, care should be taken not to categorize all keratins as contaminants because certain keratins have been identified as markers that define the degree of bladder squamous cell carcinoma differentiation [24]. Additionally, some keratins function as a diagnostic tool in early stage [25] and higher stage bladder carcinoma [26]. For example, one of these marker keratins, cytokeratin type 10 (keratin 10), is also part of our validated list of 697 proteins.

The number of 'Hypothetical proteins' is quite different between datasets. The HUPO high confidence list (795 proteins) contains 172 hypothetical entries (22%), which is far above our number of 30 hypothetical proteins (4.3%). Anderson et al. [23] report only 3 hypothetical proteins within their list of 195 confirmed proteins (1.5%). It is worth noting that the HUPO list contains immunoglobulin entries, whereas all of the Igs were lumped into one accession because of high sequence similarity (>95%) in the Anderson study. We have completely removed immunoglobulins from our list of 697 high confidence proteins for comparison purposes (see section 'Immunoglobulins' below).

### Isoforms

Some investigators omit specific isoforms, thus simplifying the picture by collapsing all protein isoforms into one entry. Anderson et al. 2004 assigned all sequences that shared a region larger than 15 amino acids and having greater than 95% sequence identity to one single entry. By doing this, it is likely that they have missed some splice variants or specific protein isoforms. As reported in the results section, we subjected all of our protein sequences to an 'All vs. All BLAST' search. After that, we verified all proteins with 95% or greater sequence identity by visual inspection to determine whether the identified peptides matched the protein sequences they were assigned to, and whether it was justified to maintain similar protein sequences as separate entities rather than joining them into one entry. Finally, we employed a peptide mapping approach to identify any remaining redundancy in our list, merging proteins together where appropriate and assigning an annotation reflecting the accuracy of the available information.

A database searching tool such as Mascot often retrieves a specific isoform for a particular protein. However, upon manual inspection, it is clear that many times the sequenced peptide(s) do not specifically distinguish between the different isoforms and thus reveal that the isoform indicated by Mascot is not correct. In our dataset, we claim identification of a specific protein isoform only if there was at least one validated peptide identified that specifically differentiated between the possible isoforms for this particular protein. We believe that the extra step of manually validating these isoforms is justified because only an accurate and detailed plasma proteome can serve as a reference for future biomarker discoveries and diagnostic applications.

Specific isoforms may not only be identified as having specific tissue preferences but they may also be involved in unique functions. The importance of specific isoforms is exemplified by certain growth factors, such as TGF-beta or enzymes such as superoxide dismutase (SOD). TGF-beta1, -beta2, and -beta3 are isoforms that share high sequence identity and play crucial and only partially overlapping roles in hematopoiesis [27], development, tumor suppression, and wound healing [28]. SOD-1 is localized in the cytosol, SOD-2 in mitochondria, and SOD-3 is extracellular, and in addition to their unique subcellular localization, all three isoforms have major and distinctive responsibilities in the response process of vascular cells to both acute and chronic oxidative stress [29]. While distinct subcellular localizations of specific isoforms are quite often known, their specific functions remain to be clarified in most cases. For example we identified the protein attractin, isoform 2 out of 5 possible isoforms. Isoform 1 is a type I membrane protein while 2–5 are secreted proteins. Isoform 2 is the major isoform in peripheral blood leukocytes, but nothing is reported regarding the function(s) of this particular isoform. For 'heat shock cognate 71 kDa protein', the 7 unique peptides that we identified can not distinguish between the two splice isoforms 1 or 2. However, isoform 2 has a proposed function as an endogenous inhibitory regulator of HSC70, via competition with co-chaperones. Tropomyosin 1 alpha chain (TPM1) occurs in 5 isoforms of which we identified isoform 2, 3, and 5. Isoform 1 is the skeletal muscle variant, isoform 2 is the smooth muscle form, and isoform 3 is the fibroblast form, while the primary localization for isoforms 4 or 5 has not been reported. Defects in TPM1 are the cause of familial hypertrophic cardiomyopathy 3 (CMH3) with a high risk of cardiac failure and sudden cardiac death. At any rate, the overall homology between isoforms quite often exceeds 95% and blindly combining sequences with high similarity defeats the detailed identification of isoforms and splice variants, which may be necessary in order to distinguish between a healthy *versus *a diseased state.

### Immunoglobulins

As stated previously, we have chosen to remove the immunoglobulins (144) from our analysis to facilitate comparison of our data with other studies. However, we feel that analysis of the immunoglobulins is important for some of the same reasons that we provide in the "Isoforms" section above. We therefore provide a supplement to this work which is available on line [see Additional file 1]. The supplement contains a list of the immunoglobulins that we identified in this study although we have not analysed this list extensively for redundancy or to confirm that the peptides isolated completely map to their assigned protein sequences.

### Cellular component

With regard to the categorization of the plasma proteins into their cellular components according to the gene ontology consortium (GoMiner, ), Anderson et al. showed that their set of 1,175 distinct proteins consisted of 27% extracellular proteins, while the other 73% were cellular proteins (membrane, nuclear, cytoplasmic etc.). The 195 proteins that were identified by at least two sources, and which Anderson called the "confirmed set of plasma proteins", showed a different distribution. In this case, 50% of the proteins were classified as 'extracellular'. Of the 46 proteins that were identified by 4/4 sources and confirmed in the present study as well, 68% were classified as 'extracellular'. The clear increase in the proportion of 'extracellular' proteins seems to come in parallel with the increased confidence of an identified plasma protein set. Chan et al. [21] subjected their 1444 proteins to a gene ontology classification as well, finding a distribution of 40.3% intracellular, 32.65% membrane, and 6.9% cell fraction; this was summarized by an approximately 80% cellular fraction *versus *a 15–20% extracellular fraction. Pieper et al. [2] classified 47.5% of their 325 distinct proteins as extracellular (38.8% as classical plasma proteins, 8.9% other extracellular fluids). The rest were classified as vesicular proteins (incl. ER, Golgi etc.), cell membrane, and intracellular proteins.

As we mentioned previously (see Results), 77% of our 697 proteins were recognized as part of the GoMiner cellular component category. Of these, 35% were classified as 'extracellular' and 65% as cellular. It is possible that the proportion of extracellular proteins would change if our entire set of identified proteins could have been classified. If the accuracy of our list can be gauged based on the proportion of extracellular proteins it contains, then the relatively high proportion of extracellular proteins in our list seems to reinforce the confidence of our identified plasma protein set.

**Figure 10:**
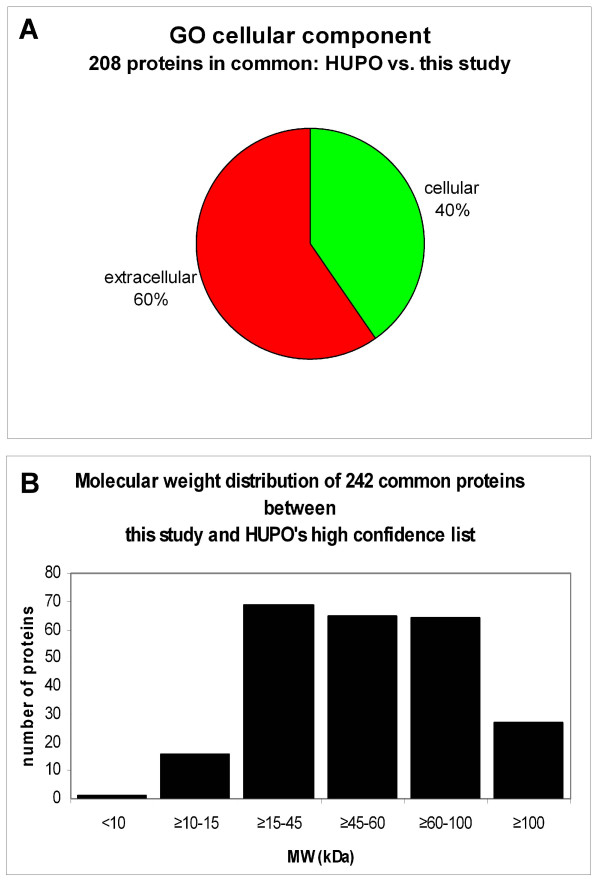
**GoMiner analysis of proteins found to be in common with this study and the HUPO study**. Panel A depicts a pie chart representation of a GoMiner analysis of the 242 proteins found to be in common between ours and the HUPO data sets. 208 of these were categorized by GoMiner to be "GO cellular component" as indicated. 98 of these proteins were categorized as cellular and 146 were categorized as extracellular, with 18 of the extracellular category being further classified as extracellular matrix proteins. Due to redundancy within the cellular and extracellular categories, the sum of the two categories was normalized to 100% for the purpose of calculating percentages. Panel B shows a histogram representing the molecular weight distribution of the 242 proteins found to be in common between ours and the HUPO data sets. The protein molecular weights were categorized as indicated in the panel before plotting.

Interestingly, if we perform a GoMiner analysis on the 242 proteins common to both our and the HUPO datasets, 227/242 (86%) of the protein IPI numbers are recognized with 208 being classified as "cellular component" by the program. As with the analysis of the BPPD dataset, some proteins appear in more than one category. GoMiner classified 98 (40%; normalized as in Results) proteins as cellular and as expected, a high percentage of proteins were classified as extracellular (146 proteins or 60%; normalized as in Results) (Figure 10, **panel A**). 18 of the extracellular proteins were classified as extracellular matrix proteins, leaving 128 "true" plasma proteins. It is more probable that independent investigators will co-identify classical plasma proteins rather than cellular proteins that might be found in plasma only as a consequence of tissue remodeling or cell death. These cellular proteins are likely to be found in the plasma only at certain points in time, so it is less plausible that independent groups will co-identify the same cellular proteins, given the disparate nature of their samples.

Comparison of the molecular weights of the 242 overlapping proteins between ours and the HUPO data sets shows a rather well-balanced distribution (Figure 10, **panel B**). Most proteins identified in our high confidence set of 697 proteins are clearly found in the 15–45 kDa range (**see **Figure 3). However, while the 242 overlapping proteins are also mainly from the range 15–45 kDa, they are very closely followed in proportion by the other MW groups with a MW ≥ 45 and up to 100 kDa. The common group of 242 proteins thus represents a cross section of plasma proteins that spans most of the possible MW range. The overlapping protein MW profile (Figure 10, **panel B**) does not resemble a MW profile as seen for a typical plasma analysis (Figure 3), or for the overlapping proteins identified within this study between the FT and Orbitrap mass spectrometers (Figure 9, **panel A**).

## Conclusion

The effort of the PPP by HUPO to standardize plasma/serum handling techniques and data analysis was a big step in the right direction. With this work we hope to contribute to this effort and to the urgent task of identifying the true nature of the human blood plasma proteome, which holds the potential for more comprehensive diagnostics and crucial biomarker discoveries. Even though the mass spectrometric methodologies and technologies currently employed are not yet able to cover the 9–12 log plasma protein concentration range, mass spectrometry-based proteomics is the 'discovery tool' of choice. Researchers are investigating alternative strategies that could help to reduce the complexity of plasma, and mass spectrometry manufacturers are continuously striving to improve the instrumentation. The latest hybrid mass spectrometer, the LTQ-Orbitrap, has been put through the acid test [30,31] and is proving itself as a very effective, high sensitivity mass spectrometer, requiring less maintenance and being less expensive than the LTQ-FTICR.

## Competing interests

The authors declare that they have no competing interests.

## Authors' contributions

SS carried out all FT-related experiments, manual data validation, drafting of the manuscript and contributed substantially to the design of the data analysis tools. GJS created all analysis tools (mainly Perl scripts) and databases and performed all bioinformatics and database-related analysis, including semi-automated data validation. GJS also contributed substantially to finalizing the manuscript. GdS performed the Orbitrap experiment. All authors read and approved the final manuscript.

## Pre-publication history

The pre-publication history for this paper can be accessed here:



## Supplementary Material

Additional file 1 List of 144 immunoglobulins excluded from our list of 697 high-confidence proteins. Click here for file

Additional file 2 List of 697 high confidence plasma proteins identified in this study. Click here for file
