# Supplementary material for: A high confidence, manually validated human blood plasma protein reference set
Source: BMC Med Genomics. 2008 Sep 15;1:41. doi: 10.1186/1755-8794-1-41 (PMC2563020; doi:10.1186/1755-8794-1-41)
Supplement: Additional file 1 — List of 144 immunoglobulins excluded from our list of 697 high-confidence proteins. [file 1755-8794-1-41-S1.doc]

**Additional file 1. List of 697 high confidence plasma proteins identified in this study.**

| **Primary accession number** | **IPI number (version 3.25)** | **Protein MW** | **Protein name** |
| --- | --- | --- | --- |
| ENSP00000351858 | IPI00477298 | 10082 | 10 kDa protein (V5-6 protein [Fragment]) |
| O75891 | IPI00290553 | 98829 | 10-formyltetrahydrofolate dehydrogenase |
| ENSP00000329622 | IPI00333234/IPI00412002 | 12340 | 12 kDa protein |
| ENSP00000353941 | IPI00414369/IPI00472961 | 12002 | 12 kDa protein |
| P31946 | IPI00216318/IPI00759832 | 27951 | 14-3-3 protein beta/alpha |
| P62258 | IPI00000816 | 29174 | 14-3-3 protein epsilon |
| Q04917 | IPI00216319 | 28088 | 14-3-3 protein eta |
| P61981 | IPI00220642 | 28171 | 14-3-3 protein gamma (Protein kinase C inhibitor protein-1) (KCIP-1) |
| P31947 | IPI00013890 | 27774 | 14-3-3 protein sigma |
| P63104 | IPI00021263 | 27745 | 14-3-3 protein zeta/delta (Protein kinase C inhibitor protein-1) (KCIP-1) |
| IPI00480016.2 | IPI00480016 | 25694 | 26 KDA PROTEIN Homo sapiens (Human). |
| O00233-1 | IPI00010860 | 24654 | 26S proteasome non-ATPase regulatory subunit 9 |
| P08195 | IPI00027493 | 57945 | 4F2 cell-surface antigen heavy chain |
| ENSP00000333488 | IPI00180730 | 50154 | 50 kDa protein |
| P19474 | IPI00018971 | 54170 | 52 kDa Ro protein |
| P08253 | IPI00027780 | 73882 | 72 kDa type IV collagenase [Precursor] |
| P11021 | IPI00003362 | 72333 | 78 kDa glucose-regulated protein [Precursor] |
| P68133 | IPI00021428 | 42051 | Actin, alpha skeletal muscle (Alpha-actin 1) |
| P60709 | IPI00021439 | 41737 | Actin, cytoplasmic 1 |
| P63261 | IPI00021440 | 41793 | Actin, cytoplasmic 2 (Gamma-actin) |
| O15144 | IPI00005161 | 34333 | Actin-related protein 2/3 complex subunit 2 (ARP2/3 complex 34 kDa subunit) (p34-ARC) |
| O15145 | IPI00005162 | 20416 | Actin-related protein 2/3 complex subunit 3 (ARP2/3 complex 21 kDa subunit) |
| P59998 | IPI00554811 | 19536 | Actin-related protein 2/3 complex subunit 4 (ARP2/3 complex 20 kDa subunit) |
| O15511 | IPI00007280 | 16189 | Actin-related protein 2/3 complex subunit 5 (ARP2/3 complex 16 kDa subunit) |
| Q9P1U1 | IPI00007068 | 47608 | Actin-related protein 3-beta |
| P07741 | IPI00218693 | 19477 | Adenine phosphoribosyltransferase |
| P23526 | IPI00012007 | 47585 | Adenosylhomocysteinase |
| P00568 | IPI00018342 | 21635 | Adenylate kinase isoenzyme 1 |
| Q01518 | IPI00008274 | 51542 | Adenylyl cyclase-associated protein 1 (CAP 1) |
| Q9HDC9 | IPI00031131 | 46480 | Adipocyte plasma membrane-associated protein |
| Q15848 | IPI00020019 | 26414 | Adiponectin [precursor] |
| Q10588 | IPI00026240 | 35724 | ADP-ribosyl cyclase 2 [Precursor] |
| P84077 | IPI00215914/IPI00215917 | 20566 | ADP-ribosylation factor 1 [mw 20,741] or ADP-ribosylation factor 3 [mw 20,645] |
| P43652 | IPI00019943 | 69069 | Afamin [Precursor] |
| Q7Z5S6 | IPI00164755 | 129288 | Alpha 2 type I collagen |
| P06733 | IPI00465248 | 47038 | Alpha enolase |
| P02763 | IPI00022429 | 23512 | Alpha-1-acid glycoprotein 1 precursor |
| P19652 | IPI00020091 | 23603 | Alpha-1-acid glycoprotein 2 [Precursor] |
| P01011 | IPI00431656/IPI00550991/IPI00411920 | 47651 | Alpha-1-antichymotrypsin [Precursor] |
| P01009 | IPI00553177/IPI00305457 | 46737 | Alpha-1-antitrypsin precursor |
| P04217 | IPI00022895/IPI00644018 | 54273 | Alpha-1B-glycoprotein [Precursor] |
| P08697 | IPI00029863 | 54566 | Alpha-2-antiplasmin [Precursor] |
| P25311 | IPI00166729 | 33872 | Alpha-2-glycoprotein 1, zinc; Zinc-alpha-2-glycoprotein [Precursor] (Zn-alpha-2-glycoprotein) (Zn- alpha-2-GP) |
| P02765 | IPI00022431 | 39325 | Alpha-2-HS-glycoprotein [Precursor] |
| P01023 | IPI00478003 | 163278 | Alpha-2-macroglobulin [Precursor] |
| P12814 | IPI00013508/IPI00013808 | 103058 | Alpha-actinin 1 |
| P19961 | IPI00021447 | 57710 | Alpha-amylase 2B [Precursor] |
| Q3LID0 | IPI00745745 | 53689 | Alpha-L-fucosidase |
| P54802 | IPI00008787 | 82167 | Alpha-N-acetylglucosaminidase [Precursor] |
| P37840 | IPI00024107 | 14460 | Alpha-synuclein, Splice isoform 1, 2, or 3 |
| P02760 | IPI00022426 | 39000 | AMBP protein [Precursor] |
| P15144 | IPI00221224 | 109380 | Aminopeptidase N |
| Q6FG67 | IPI00552578 | 13532 | Amyloid protein A [Fragment] |
| P03950 | IPI00008554 | 16550 | Angiogenin [Precursor] |
| Q9Y5C1 | IPI00004957 | 53637 | Angiopoietin-related protein 3 [Precursor] |
| P22966 | IPI00028147 | 83331 | Angiotensin-converting enzyme, testis-specific isoform [Precursor] |
| P01019 | IPI00032220/IPI00335085 | 53154 | Angiotensinogen precursor |
| P58335-2 | IPI00036552 | 42893 | Anthrax toxin receptor 2 [Precursor] |
| P49913 | IPI00292532 | 19301 | Antibacterial protein FALL-39 [Precursor] |
| P01008 | IPI00032179 | 52603 | Antithrombin-III [Precursor] |
| P02647 | IPI00021841 | 30778 | Apolipoprotein A-I [Precursor] |
| P02652 | IPI00382587/IPI00021854 | 11175 | Apolipoprotein A-II [Precursor] |
| ENSP00000350425 | IPI00304273 | 45399 | Apolipoprotein A-IV [Precursor] (Apo-AIV) |
| Q6Q788 | IPI00465378 | 41213 | Apolipoprotein A-V [Precursor] |
| P04114 | IPI00022229 | 515563 | Apolipoprotein B-100 [Precursor] |
| P02654 | IPI00021855 | 9332 | Apolipoprotein C-I [Precursor] |
| P02655 | IPI00021856 | 11284 | Apolipoprotein C-II precursor |
| P02656 | IPI00657670/IPI00021857 | 10852 | Apolipoprotein C-III [Precursor] |
| P55056 | IPI00022731 | 14553 | Apolipoprotein C-IV [Precursor] |
| P05090 | IPI00006662 | 21276 | Apolipoprotein D [Precursor] |
| P02649 | IPI00021842 | 36154 | Apolipoprotein E [Precursor] |
| NP_001629 | IPI00299435 | 35400 | Apolipoprotein F [Precursor] |
| O95445 | IPI00030739/IPI00645213 | 21253 | Apolipoprotein M |
| P08519 | IPI00029168 | 501320 | Apolipoprotein(a) [Precursor] |
| O14791-2 | IPI00186903/IPI00514475 | 45871 | Apolipoprotein-L1 [Precursor], splice isoform 2 |
| P05089 | IPI00291560/IPI00038356 | 34735 | Arginase-1 (EC 3.5.3.1), splice isoform 1 or 2 |
| P17174 | IPI00219029 | 46116 | Aspartate aminotransferase, cytoplasmic |
| P25705 | IPI00440493 | 59751 | ATP synthase alpha chain, mitochondrial [Precursor] |
| O75882-2 | IPI00027235/IPI00162735 | 141429 | Attractin [Precursor], Splice isoform 2 |
| P98160 | IPI00024284 | 468826 | Basement membrane-specific heparan sulfate proteoglycan core protein [Precursor] |
| P13929 | IPI00218474 | 46856 | Beta enolase |
| Q13885 | IPI00013475 | 49907 | Beta tubulin (Tubulin beta 2, OTTHUMP00000015956) |
| P02749 | IPI00298828 | 38298 | Beta-2-glycoprotein I [Precursor] |
| P61769 | IPI00004656 | 13715 | Beta-2-microglobulin [Precursor] |
| Q9HBI1 | IPI00043083 | 41714 | Beta-parvin |
| P43251 | IPI00218413 | 58913 | Biotinidase [Precursor] |
| P07738 | IPI00215979 | 29874 | Bisphosphoglycerate mutase |
| Q13867 | IPI00219575 | 52562 | Bleomycin hydrolase |
| Q9UKN4 | IPI00234667/IPI00550792 | 61747 | Bridging integrator-2 or Breast cancer associated protein BRAP1 (Q9UBW5) |
| Q96CX2 | IPI00060715 | 35701 | BTB/POZ domain-containing protein KCTD12 |
| P02741-1 | IPI00022389 | 25039 | C-reactive protein [Precursor], Splice isoform 1 |
| P04003 | IPI00021727 | 67033 | C4b-binding protein alpha chain [Precursor] |
| P20851 | IPI00025862 | 28357 | C4b-binding protein beta chain [Precursor] |
| P55290 | IPI00024046 | 78287 | Cadherin-13 [Precursor] |
| P06703 | IPI00027463 | 10180 | Calcyclin |
| Q05682 | IPI00014516 | 93251 | Caldesmon, Splice Isoform 1, 2, 3, 4, or 5 |
| P05109 | IPI00007047 | 10835 | Calgranulin A |
| P06702 | IPI00027462 | 13242 | Calgranulin B |
| P27482 | IPI00216984 | 16760 | Calmodulin-like protein 3 |
| Q9NZT1 | IPI00021536 | 15921 | Calmodulin-like protein 5 |
| P04632 | IPI00025084 | 28316 | Calpain small subunit 1 |
| Q99439 | IPI00015262 | 33566 | Calponin H2, smooth muscle or Calponin 2 isoform a variant |
| P27797 | IPI00020599 | 48142 | Calreticulin [Precursor] |
| O43852 | IPI00014537 | 37107 | Calumenin [Precursor] Splice Isoform 1 or 2 |
| Q92887 | IPI00023868 | 174191 | Canalicular multispecific organic anion transporter 1 |
| P00918 | IPI00218414 | 29115 | Carbonic anhydrase 2 (EC 4.2.1.1) (Carbonate dehydratase II) (CA-II) (Carbonic anhydrase C) |
| P07451 | IPI00216983 | 29440 | Carbonic anhydrase 3 |
| P00915 | IPI00215983 | 28739 | Carbonic anhydrase I |
| P16152 | IPI00295386 | 30244 | Carbonyl reductase [NADPH] 1 |
| Q96IY4 | IPI00329775/IPI00293057 | 48412 | Carboxypeptidase B2 [Precursor] (EC 3.4.17.20) (Thrombin-activatable fibrinolysis inhibitor) |
| P15169 | IPI00010295 | 52286 | Carboxypeptidase N catalytic chain [Precursor] |
| NP_116038 | IPI00064667 | 56692 | Carnosinase 1 (carnosine dipeptidase 1, Beta-Ala-His dipeptidase) |
| Q9NQ79 | IPI00451624 | 71421 | Cartilage acidic protein 1 [Precursor], Splice Isoform 1, 2, or 3 |
| P49747 | IPI00028030 | 82832 | Cartilage oligomeric matrix protein [Precursor] |
| P31944 | IPI00013885 | 27680 | Caspase-14 [Precursor] |
| P04040 | IPI00465436 | 59625 | Catalase |
| P07339 | IPI00011229 | 44552 | Cathepsin D [Precursor] |
| P25774 | IPI00299150 | 37496 | Cathepsin S [Precursor] |
| Q9UBR2 | IPI00002745 | 33868 | Cathepsin Z [Precursor] |
| P11717 | IPI00289819 | 274276 | Cation-independent mannose-6-phosphate receptor [Precursor] |
| Q5SYA8 | IPI00152540 | 161689 | CD109 antigen [Precursor] |
| O43866 | IPI00025204 | 38088 | CD5 antigen-like [Precursor] |
| P21926 | IPI00215997 | 25285 | CD9 antigen |
| P14209-1 | IPI00253036 | 18848 | CD99 antigen [Precursor] |
| Q6ZP84 | IPI00418663 | 53025 | CDNA FLJ26317 fis, clone DMC09625 |
| Q96NS7 | IPI00043862 | 64007 | CDNA FLJ30149 fis, clone BRACE2000280, weakly similar to MNN4 PROTEIN |
| Q8NA87 | IPI00103481/IPI00329306 | 44698 | CDNA FLJ35741 fis, clone TESTI2004163, moderately similar to Mus musculus type II cytokeratin |
| NP_006605 | IPI00299059 | 136698 | Cell adhesion molecule with homology to L1CAM precursor [Homo sapiens] |
| P60953-2 | IPI00016786/IPI00007189 | 21259 | Cell division control protein 42 homolog, Splice isoform 2 |
| P43121-1 | IPI00016334/IPI00445227 | 71608 | Cell surface glycoprotein MUC18 [Precursor], splice isoform 1 (Melanoma-associated antigen MUC18) (CD146 antigen) |
| Q8WY20 | IPI00103595 | 350937 | Centrosome-associated protein 350 |
| P00450 | IPI00017601 | 122205 | Ceruloplasmin [Precursor] |
| O00299 | IPI00010896 | 26792 | Chloride intracellular channel protein 1 |
| P11597 | IPI00006173 | 54770 | Cholesteryl ester transfer protein precursor |
| P06276 | IPI00025864 | 68418 | Cholinesterase [Precursor] |
| O15335 | IPI00014592 | 40488 | Chondroadherin [Precursor] |
| Q9BUN1 | IPI00550533 | 36770 | Chromosome 1 open reading frame 56 |
| Q00610 | IPI00024067 | 191484 | Clathrin heavy chain 1, splice isoform 1 or 2 |
| P09496 | IPI00216393 | 27077 | Clathrin light chain A (Lca) |
| P10909 | IPI00291262 | 52495 | Clusterin [Precursor] |
| Q14019 | IPI00017704 | 15814 | Coactosin-like protein |
| P00740 | IPI00296176 | 51748 | Coagulation factor IX [Precursor] (Christmas factor) |
| P12259 | IPI00022937/IPI00478809 | 251672 | Coagulation factor V [Precursor] |
| P00742 | IPI00019576 | 54732 | Coagulation factor X [Precursor] (Stuart factor) |
| P03951-1 | IPI00008556 | 70109 | Coagulation factor XI [Precursor] |
| P00748 | IPI00019581 | 67818 | Coagulation factor XII [Precursor] (Hageman factor) |
| P00488 | IPI00297550 | 83136 | Coagulation factor XIII A chain [Precursor] |
| P05160 | IPI00007240 | 75492 | Coagulation factor XIII B chain [Precursor] |
| P23528 | IPI00012011 | 18371 | Cofilin-1, non-muscle isoform |
| P02452 | IPI00297646 | 138884 | Collagen alpha 1(I) chain [Precursor] |
| P39059 | IPI00295414 | 141931 | Collagen alpha 1(XV) chain precursor |
| P20908 | IPI00477611 | 183560 | Collagen alpha-1(V) chain [Precursor] |
| P39060-1 | IPI00022822 | 153828 | Collagen alpha-1(XVIII) chain [Precursor] |
| ENSP00000295550 | IPI00022200 | 343670 | Collagen alpha-3(VI) chain precursor. |
| Q3SYH6 | IPI00007917 | 30705 | Collectin sub-family member 10 |
| Q9BWP8 | IPI00031490 | 28665 | Collectin sub-family member 11 |
| P02745 | IPI00022392 | 26017 | Complement C1q subcomponent, A chain [Precursor] |
| P02746 | IPI00477992 | 26459 | Complement C1q subcomponent, B chain [Precursor] |
| P02747 | IPI00022394 | 25774 | Complement C1q subcomponent, C chain [Precursor] |
| Q9BXJ4-1 | IPI00008860 | 26994 | Complement C1q tumor necrosis factor-related protein 3 [Precursor] |
| P00736 | IPI00479867/IPI00170999/IPI00296165 | 80174 | Complement C1r subcomponent [Precursor] (EC 3.4.21.41) |
| P09871 | IPI00017696 | 76685 | Complement C1s subcomponent [Precursor] |
| P06681 | IPI00303963 | 83268 | Complement C2 [Precursor] |
| ENSP00000245907 | IPI00164623/IPI00739237 | 187306 | Complement C3 precursor [Contains: Complement C3 beta chain; Complement C3 alpha chain; C3a anaphylatoxin; Complement C3b alpha' chain; Complement C3c fragment; Complement C3dg fragment; Complement C3g fragment; Complement C3d fragment; Complement C3f fra Source: Uniprot/SWISSPROT P01024 |
| P01024 | IPI00164623/IPI00738274 | 187164 | Complement C3 [Precursor] |
| P0C0L4 | IPI00032258/IPI00654875 | 192772 | Complement C4-A - Homo sapiens (Human). |
| P01031 | IPI00032291/IPI00169407 | 188331 | Complement C5 [Precursor] |
| Q53GX9 | IPI00009793 | 53541 | Complement component 1, r subcomponent-like variant [Fragment] |
| Q5JNX2 | IPI00555805/IPI00643525 | 192743 | Complement component 4A |
| NP_000583 | IPI00418163 | 192798 | Complement component 4B preproprotein |
| Q9NPY3 | IPI00299485 | 68560 | Complement component C1q receptor [Precursor] |
| Q6U2E9 | IPI00418163 | 192752 | Complement component C4B, C4B1 |
| P13671 | IPI00009920 | 104844 | Complement component C6 [Precursor] |
| P10643 | IPI00296608 | 93518 | Complement component C7 [Precursor] |
| P07357 | IPI00011252 | 65163 | Complement component C8 alpha chain [Precursor] |
| P07358 | IPI00294395 | 67047 | Complement component C8 beta chain [Precursor] |
| P07360 | IPI00011261 | 22220 | Complement component C8 gamma chain [Precursor] |
| P02748 | IPI00022395 | 63174 | Complement component C9 [Precursor] |
| P00751-1 | IPI00019591 | 85533 | Complement factor B [Precursor], Splice isoform 1 |
| P00746 | IPI00019579 | 27004 | Complement factor D [Precursor] |
| P08603-1 | IPI00029739 | 139071 | Complement factor H [Precursor], splice isoform 1 |
| Q5TFM6 | IPI00011264/IPI00167093 | 37651 | Complement factor H-related protein 1 |
| Q03591 | IPI00011264 | 37662 | Complement factor H-related protein 1 [Precursor] |
| P36980 | IPI00006154 | 30651 | Complement factor H-related protein 2 [Precursor], Splice Isoform 1 or 2 |
| Q02985 | IPI00027507 | 37323 | Complement factor H-related protein 3 [Precursor] |
| Q92496 | IPI00021578 | 37325 | Complement factor H-related protein 4 [Precursor] |
| Q9BXR6 | IPI00006543 | 64419 | Complement factor H-related protein 5 [Precursor] (FHR5_HUMAN) |
| P05156 | IPI00291867 | 65720 | Complement factor I [Precursor] |
| Q96RS4 | IPI00290283 | 81860 | Complement factor MASP-3 |
| P48740 | IPI00299307 | 79259 | Complement-activating component of Ra-reactive factor [Precursor] |
| Q12860-1 | IPI00029751 | 113321 | Contactin-1 [Precursor] |
| Q15363 | IPI00016608 | 22761 | Cop-coated vesicle membrane protein p24 [Precursor] |
| Q15517 | IPI00386809 | 51495 | Corneodesmosin [Precursor] |
| P35321 | IPI00017987 | 9883 | Cornifin A |
| P31146 | IPI00010133 | 51026 | Coronin-1A |
| P08185 | IPI00027482 | 45141 | Corticosteroid-binding globulin [Precursor] |
| P24387 | IPI00306844 | 36144 | Corticotropin-releasing factor-binding protein [Precursor] |
| P06732 | IPI00027487 | 43101 | Creatine kinase M-type |
| P01040 | IPI00032325 | 11006 | Cystatin A |
| P04080 | IPI00021828 | 11140 | Cystatin B |
| P01034 | IPI00032293 | 15799 | Cystatin C [Precursor] |
| Q15828 | IPI00019954 | 16511 | Cystatin M [Precursor] |
| P21291 | IPI00442073 | 20436 | Cysteine and glycine-rich protein 1 |
| P54108 | IPI00004798 | 27630 | Cysteine-rich secretory protein 3 [Precursor] |
| O95678 | IPI00005859 | 59504 | Cytokeratin type II |
| P30046 | IPI00293867 | 12581 | D-dopachrome decarboxylase |
| Q9UGM3-4 | IPI00418512 | 166502 | Deleted in malignant brain tumors 1 protein [Precursor] |
| P13716 | IPI00010314 | 36295 | Delta-aminolevulinic acid dehydratase |
| P81605 | IPI00027547 | 11284 | Dermcidin [Precursor] |
| Q9HB00 | IPI00007425 | 93835 | Desmocollin 1b |
| Q02487 | IPI00025846 | 99962 | Desmocollin-2 [Precursor], Splice Isoform 1 or 2 |
| Q14574-1 | IPI00031549 | 99969 | Desmocollin-3 [Precursor] |
| Q02413 | IPI00025753 | 113716 | Desmoglein-1 [Precursor] |
| Q14126 | IPI00028931 | 122385 | Desmoglein-2 [Precursor] |
| P15924-1 | IPI00217182/IPI00013933 | 331774 | Desmoplakin |
| P60981 | IPI00031045 | 18375 | Destrin (Actin-depolymerizing factor, ADF) |
| Q01459 | IPI00007778 | 43760 | Di-N-acetylchitobiase [Precursor] |
| P27487 | IPI00018953 | 88279 | Dipeptidyl peptidase 4 |
| Q99497 | IPI00298547 | 19891 | DJ-1 protein (Oncogene DJ1) |
| P09172 | IPI00171678 | 67613 | Dopamine beta-hydroxylase [Precursor] |
| P51452 | IPI00018671 | 20478 | Dual specificity protein phosphatase 3 |
| Q9UII8 | IPI00000513 | 90942 | E-cadherin |
| Q13822-1 | IPI00156171 | 99004 | Ectonucleotide pyrophosphatase/phosphodiesterase 2 |
| Q7Z754 | IPI00157365/IPI00293026 | 97013 | EFTUD1 protein |
| Q9HBW9 | IPI00374007 | 77825 | EGF, latrophilin and seven transmembrane domain-containing protein 1 [Precursor] |
| Q12805 | IPI00029658 | 54641 | EGF-containing fibulin-like extracellular matrix protein 1 [Precursor], Splice Isoform 1, 2, 3, or 4 |
| P24534 | IPI00178440 | 24633 | Elongation factor 1 b (Eukaryotic translation Eta 2) |
| P68104 | IPI00014424/IPI00025447 | 50141 | Elongation factor 1-alpha 1 (EF-1-alpha-1) (Elongation factor 1 A-1) (eEF1A-1) (Elongation factor Tu |
| P13639 | IPI00186290 | 95207 | Elongation factor 2 |
| Q9Y6C2 | IPI00013079 | 106696 | EMILIN 1 precursor |
| P14625 | IPI00027230 | 92469 | Endoplasmin [Precursor] |
| Q9UNN8 | IPI00009276 | 26671 | Endothelial protein C receptor [Precursor] |
| P27105 | IPI00219682 | 31600 | Erythrocyte band 7 integral membrane protein |
| Q9GZV4 | IPI00006935 | 16793 | Eukaryotic translation initiation factor 5AII |
| Q9UBQ6 | IPI00002732 | 37466 | Exostosin-like 2 |
| Q9GZZ8 | IPI00020487 | 14246 | Extracellular glycoprotein lacritin precursor |
| Q16610 | IPI00003351 | 60674 | Extracellular matrix protein 1 [Precursor] |
| P08294 | IPI00027827 | 25881 | Extracellular superoxide dismutase [Cu-Zn] [Precursor] |
| P52907 | IPI00005969 | 32923 | F-actin capping protein alpha-1 subunit |
| P47755 | IPI00412598/IPI00026182 | 32818 | F-actin capping protein alpha-2 subunit |
| P47756 | IPI00026185 | 31219 | F-actin capping protein beta subunit, Splice isoform 1 or 2 |
| P14324 | IPI00101405 | 40533 | Farnesyl diphosphate synthase |
| P15090 | IPI00215746 | 14588 | Fatty acid-binding protein, adipocyte |
| NP_001435 | IPI00007797 | 15164 | Fatty acid-binding protein, epidermal |
| P07148 | IPI00010290 | 14208 | Fatty acid-binding protein, liver |
| P02792 | IPI00397828/IPI00375676 | 19889 | Ferritin light chain (Ferritin L subunit) |
| Q9UGM5 | IPI00005439 | 42094 | Fetuin-B [Precursor] |
| P02671-1 | IPI00021885 | 94973 | Fibrinogen alpha chain [Precursor] (Fibrinogen alpha/alpha-E chain precursor), Splice isoform Alpha-E |
| P02675 | IPI00298497 | 55928 | Fibrinogen beta chain [Precursor] |
| P02679-2 | IPI00167009/IPI00219713/IPI00021891 | 49496 | Fibrinogen gamma chain [Precursor], splice isoform 2 (isoform Gamma-A) |
| Q08830 | IPI00303482 | 36392 | Fibrinogen-like protein 1 [Precursor] (Hepatocyte-derived fibrinogen-related protein 1) |
| NP_997643 | IPI00470919/IPI00022418/IPI00414283/  IPI00556632 | 256512 | fibronectin 1, isoform CRA_n, isoform CRA_j, isoform CRA_h, isoform CRA_m, isoform 4, isoform 5, isoform 3 [Homo sapiens] |
| P02751 | IPI00022418 | 262606 | Fibronectin precursor, Splice Isoform 1, 3, 5, 7, 8, 9, or 10 |
| P02751-8 | IPI00339228/IPI00411462 | 252793 | Fibronectin [Precursor], Splice isoform 8 |
| P23142-4 | IPI00296537 | 74462 | Fibulin-1 precursor, Splice isoform 4 |
| P23142-1 | IPI00218803/IPI00296534 | 77261 | Fibulin-1 [Precursor], Splice Isoform 1 |
| Q15485 | IPI00017530 | 34019 | Ficolin 2 [Precursor] |
| O75636-1 | IPI00293925/IPI00419744 | 32903 | Ficolin-3 [Precursor], splice isoform 1 (Collagen/fibrinogen domain-containing protein 3) (Hakata antigen) |
| P20930 | IPI00026256 | 435171 | Filaggrin |
| Q5HY53 | IPI00302592/IPI00333541 | 280018 | Filamin A, alpha (actin binding protein 280) |
| P30043 | IPI00219910 | 21988 | Flavin reductase |
| Q8NF17 | IPI00168728 | 56111 | FLJ00385 protein [Fragment] |
| P09467 | IPI00073772 | 36683 | Fructose-1,6-bisphosphatase 1 |
| P04075 | IPI00465439/IPI00454862 | 39289 | Fructose-bisphosphate aldolase A (Muscle-type aldolase, Lung cancer antigen NY-LU-1) |
| P05062 | IPI00218407 | 39342 | Fructose-bisphosphate aldolase B |
| Q86SX2 | IPI00328493 | 15573 | Full-length cDNA clone CS0DL004YM19 of B cells (Ramos cell line) of Homo sapiens [Fragment] |
| P16930 | IPI00031708 | 46374 | Fumarylacetoacetase |
| P17931 | IPI00465431 | 26057 | Galectin-3 |
| Q08380 | IPI00023673 | 65331 | Galectin-3 binding protein [Precursor] |
| P47929 | IPI00219221 | 14944 | Galectin-7 |
| P09104 | IPI00216171 | 47137 | Gamma enolase (EC 4.2.1.11) (2-phospho-D-glycerate hydro-lyase) (Neural enolase) (Neuron-specific enolase) (NSE) (Enolase 2) |
| Q92820 | IPI00023728 | 35964 | Gamma-glutamyl hydrolase [Precursor] |
| Q96QA5 | IPI00166200 | 49365 | Gasdermin |
| Q9NS71 | IPI00021342 | 20331 | Gastrokine-1 [Precursor] |
| P06396 | IPI00377087/IPI00026314 | 85698 | Gelsolin [Precursor], plasma (Actin-depolymerizing factor) |
| O60234 | IPI00028414 | 16801 | Glia maturation factor gamma |
| P35754 | IPI00219025 | 11645 | Glutaredoxin-1 |
| P46439 | IPI00419235 | 25562 | Glutathione S-transferase Mu 5 |
| P09211 | IPI00219757 | 23225 | Glutathione S-transferase P |
| P78417 | IPI00019755 | 27566 | Glutathione transferase omega 1 |
| P04406 | IPI00219018 | 35922 | Glyceraldehyde-3-phosphate dehydrogenase (EC 1.2.1.12) (GAPDH) |
| Q14749 | IPI00215925 | 32611 | Glycine N-methyltransferase |
| P11216 | IPI00004358 | 96565 | Glycogen phosphorylase, brain form |
| P11217 | IPI00218130 | 96961 | Glycogen phosphorylase, muscle form |
| Q03870 | IPI00043073 | 12704 | Glycophorin Erik (STA) [Precursor] |
| Q9H4G4 | IPI00007067 | 17087 | Golgi-associated plant pathogenesis-related protein 1 (Golgi-associated PR-1 protein) (GAPR-1) (Glioma pathogenesis-related protein 2) (GliPR 2) |
| Q7M4S4 | IPI00394712 | 2046 | Granulocyte inhibitory protein |
| Q8IWJ2 | IPI00005631/IPI00333197 | 195911 | GRIP and coiled-coil domain-containing protein 2, Splice Isoform 1 or 2 |
| Q9NR31 | IPI00002149/IPI00015954 | 22367 | GTP-binding protein SAR1a |
| P00738 | IPI00431645/IPI00641737/IPI00478493 | 45205 | Haptoglobin [Precursor] |
| Q92659 | IPI00477597 | 43077 | Haptoglobin-related protein [Precursor] |
| P34931 | IPI00301277 | 70375 | Heat shock 70 kDa protein 1L |
| P11142 | IPI00003865/IPI00397340 | 70898 | Heat shock cognate 71 kDa protein, Splice isoform 1 or 2 |
| P04792 | IPI00025512 | 22783 | Heat-shock protein beta-1 |
| P69905 | IPI00410714 | 15126 | Hemoglobin alpha chain (Hemoglobin subunit alpha) (Alpha-globin) |
| P68871 | IPI00654755 | 15867 | Hemoglobin beta chain (Hemoglobin subunit beta) (Beta-globin) |
| P02042 | IPI00473011/IPI00654755 | 15924 | Hemoglobin delta chain |
| P69891 | IPI00749035/IPI00220706 | 16009 | Hemoglobin subunit gamma-1 |
| P02790 | IPI00022488 | 51676 | Hemopexin [Precursor] |
| P05546 | IPI00292950 | 57071 | Heparin cofactor II [Precursor] |
| Q04756 | IPI00029193 | 70682 | Hepatocyte growth factor activator [Precursor] |
| P26927 | IPI00385496/IPI00292218 | 80380 | Hepatocyte growth factor-like protein [Precursor] |
| P19367 | IPI00018246 | 102503 | Hexokinase, type I, Splice isoform 1, 2, 3, or 4 |
| Q14520 | IPI00041065 | 62672 | HGF activator like protein |
| Q6UXB8 | IPI00301143 | 49471 | HGSC289 |
| P37235 | IPI00219344 | 22182 | Hippocalcin-like protein 1 |
| P04196 | IPI00022371 | 59579 | Histidine-rich glycoprotein [Precursor] |
| Q92769 | IPI00289601 | 55325 | Histone deacetylase 2 |
| P16403 | IPI00217465 | 21233 | Histone H1.2 |
| P10412 | IPI00217467 | 21734 | Histone H1.4 |
| P16401 | IPI00217468 | 22449 | Histone H1.5 |
| Q99878 | IPI00552873/IPI00031562/IPI00018278 | 13805 | Histone H2A type 1-J |
| Q93079 | IPI00018534/IPI00003935/IPI00303133 | 13761 | Histone H2B type 1-H |
| Q71DI3 | IPI00171611 | 15388 | Histone H3 |
| P62805 | IPI00453473 | 11236 | Histone H4 |
| P01892 | IPI00644631 | 40922 | HLA class I histocompatibility antigen, A-2 alpha chain [Precursor] |
| Q9NSD6 | IPI00552267 | 11306 | Homo sapiens This CDS feature is included to show the translation of the corresponding V_region. Presently translation qualifiers on V_region features are illegal. [Fragment] |
| Q86YZ3 | IPI00739504/IPI00398625 | 282391 | Hornerin |
| Q6P5T6 | IPI00023549 | 18986 | HSPC159 (Galectin, galactose-binding lectin family protein) |
| NP_005317 | IPI00003933 | 33806 | hydroxyacyl glutathione hydrolase isoform 1 [Homo sapiens]. |
| Q96EK7 | IPI00335946 | 103784 | Hypothetical KIAA1838 protein, Novel protein |
| Q7Z2U7 | IPI00784519 | 25015 | Hypothetical protein |
| Q6P6C4 | IPI00784807/IPI00399007 | 51325 | Hypothetical protein |
| Q96HD6 | IPI00063022 | 7218 | Hypothetical protein |
| Q8N355 | IPI00154742 | 24793 | Hypothetical protein |
| Q6DHW4 | IPI00784711 | 25108 | Hypothetical protein |
| Q8TBC9 | IPI00550162 | 24867 | Hypothetical protein |
| Q6PIH4 | IPI00478600 | 25871 | Hypothetical protein |
| Q6PIH6 | IPI00419424 | 26235 | Hypothetical protein |
| Q86T72 | IPI00297160 | 39388 | Hypothetical protein DKFZp451K1918 |
| Q6MZU6 | IPI00426051 | 51099 | Hypothetical protein DKFZp686C15213 |
| Q7Z3Q0 | IPI00375843 | 54176 | Hypothetical protein DKFZp686J1375 |
| Q6N0B3 | IPI00334627/IPI00418169 | 40353 | Hypothetical protein DKFZp686P03159 |
| Q9NTK6 | IPI00382470/IPI00335118/IPI00334775 | 84844 | Hypothetical protein DKFZp761K0511 (Heat shock protein HSP 90-beta) |
| Q8TEE1 | IPI00256429 | 96021 | Hypothetical protein FLJ00256 protein [Fragment] |
| Q8NF52 | IPI00302592 | 281430 | Hypothetical protein FLJ00343 protein [Fragment] |
| Q9NVH2 | IPI00743871 | 106834 | Hypothetical protein FLJ10736 |
| Q8TC09 | IPI00514795 | 55446 | Hypothetical protein FLJ23033 protein |
| Q6ZP87 | IPI00442911 | 22226 | Hypothetical protein FLJ26266 |
| Q8N274 | IPI00021812 | 86052 | Hypothetical protein FLJ33834 |
| Q6ZW64 | IPI00446503 | 53321 | Hypothetical protein FLJ41552 |
| O75055 | IPI00002221 | 226008 | Hypothetical protein KIAA0467 protein [Fragment] |
| Q9Y2H3 | IPI00646328 | 173464 | Hypothetical protein KIAA0967 protein |
| Q9ULK3 | IPI00187002 | 146178 | Hypothetical protein KIAA1217 protein [Fragment] |
| Q9ULE7 | IPI00306048 | 68109 | Hypothetical protein KIAA1273 protein [Fragment] |
| XP_496159 | IPI00470653/IPI00455296/IPI00183390 | 163728 | Hypothetical protein KIAA1501 protein, PREDICTED similarity |
| Q86XI8 | IPI00373968 | 64179 | Hypothetical protein LOC374920 |
| NP_787066 | IPI00449202 | 13213 | Hypothetical protein LOC90925 |
| XP_374290 | IPI00375052 | 13209 | Hypothetical protein XP_374290, Predicted |
| Q8TBD0 | IPI00152189 | 14012 | Hypothetical protein [Fragment] |
| Q5D862 | IPI00397801 | 248072 | Ifapsoriasin (Filaggrin 2) |
| Q14974 | IPI00001639 | 97170 | Importin beta-1 subunit |
| P55103 | IPI00023314 | 38238 | Inhibin beta C chain [Precursor] |
| P17936 | IPI00018305 | 31660 | Insulin-like growth factor binding protein 3 [Precursor] |
| P22692 | IPI00305380 | 27934 | Insulin-like growth factor binding protein 4 precursor |
| P24592 | IPI00029235 | 25323 | Insulin-like growth factor binding protein 6 precursor |
| P35858 | IPI00020996 | 66035 | Insulin-like growth factor binding protein complex acid labile chain precursor |
| P01343 | IPI00001610 | 17026 | Insulin-like growth factor IA precursor |
| P01344-1 | IPI00001611 | 20140 | Insulin-like growth factor II [Precursor], Splice Isoform 1 |
| P18065 | IPI00297284 | 35138 | Insulin-like growth factor-binding protein 2 [Precursor] |
| P24593 | IPI00029236 | 30570 | Insulin-like growth factor-binding protein 5 [Precursor] |
| Q16270 | IPI00016915 | 29130 | Insulin-like growth factor-binding protein 7 [Precursor] |
| P17301 | IPI00013744 | 129295 | Integrin alpha-2 [Precursor] |
| P23229 | IPI00010697 | 126619 | Integrin alpha-6 [Precursor], Splice isoform 1, 2, 3, 4, 5, or 6 |
| P08514-1 | IPI00295976/IPI00218628 | 113391 | Integrin alpha-IIb [Precursor], Splice isoform 1 |
| P05106 | IPI00220350 | 86269 | Integrin beta-3 [Precursor], Splice isoform 1, 2, or 3 |
| Q8WUM6 | IPI00645194 | 88415 | Integrin, beta 1 |
| Q13418 | IPI00013219 | 51419 | Integrin-linked protein kinase 1 |
| Q3B7H5 | IPI00028413 | 99849 | Inter-alpha (Globulin) inhibitor H3 - Homo sapiens (Human). |
| P19827 | IPI00292530/IPI00383338 | 101389 | Inter-alpha-trypsin inhibitor heavy chain H1 [Precursor] (ITI heavy chain H1) (Inter-alpha-inhibitor heavy chain 1) (Inter-alpha-trypsin inhibitor complex component III) (Serum-derived hyaluronan-associated protein) (SHAP) |
| P19823 | IPI00289083/IPI00305461 | 106436 | Inter-alpha-trypsin inhibitor heavy chain H2 [Precursor] |
| Q14624-1 | IPI00294193 | 103358 | Inter-alpha-trypsin inhibitor heavy chain H4 [Precursor], Splice isoform 1 |
| Q14624-2 | IPI00218192 | 101242 | Inter-alpha-trypsin inhibitor heavy chain H4 [Precursor], Splice isoform 2 |
| P13598 | IPI00009477 | 30654 | Intercellular adhesion molecule 2 [Precursor] |
| P05362 | IPI00008494 | 57826 | Intercellular adhesion molecule-1 [Precursor] |
| Q9NPH3-1 | IPI00031789 | 65418 | Interleukin-1 receptor accessory protein [Precursor] |
| P40189 | IPI00297124 | 103523 | Interleukin-6 receptor subunit beta [Precursor], isoform 1 or 2 |
| P21399 | IPI00008485 | 98399 | Iron-responsive element binding protein 1 |
| P14923 | IPI00554711 | 81498 | Junction plakoglobin |
| P29622 | IPI00328609 | 48542 | Kallistatin [Precursor] |
| NP_000412 | IPI00009865 | 58827 | keratin 10 [Homo sapiens] |
| Q4VAQ2 | IPI00021304 | 65433 | Keratin 2 |
| Q7RTT2 | IPI00166205 | 56965 | Keratin 5b |
| Q3SY84 | IPI00061200 | 57222 | Keratin 71 or Keratin 6 irs |
| Q15323 | IPI00032513 | 47232 | Keratin, type I cuticular Ha1 |
| Q14532 | IPI00291540 | 50319 | Keratin, type I cuticular Ha2 |
| Q14525 | IPI00031423 | 46214 | Keratin, type I cuticular Ha3-II |
| P13646-1 | IPI00009866 | 49586 | Keratin, type I cytoskeletal 13 |
| P02533 | IPI00384444 | 51490 | Keratin, type I cytoskeletal 14 |
| gi|125081 | IPI00290077 | 49168 | Keratin, type I cytoskeletal 15 (Cytokeratin-15) (CK-15) (Keratin-15) (K15). |
| P08779 | IPI00164375/IPI00217963 | 51137 | Keratin, type I cytoskeletal 16 (Cytokeratin 16) |
| Q04695 | IPI00450768 | 47975 | Keratin, type I cytoskeletal 17 |
| Q96FV1 | IPI00479145 | 45898 | Keratin, type I cytoskeletal 19 |
| NP_000217 | IPI00019359 | 62064 | Keratin, type I cytoskeletal 9 (Keratin-9) |
| Q14533 | IPI00182655/IPI00182654 | 54972 | Keratin, type II cuticular Hb1 or Hb6 |
| Q9NSB4 | IPI00300053 | 56683 | Keratin, type II cuticular Hb2 |
| Q9NSB2 | IPI00300052 | 64896 | Keratin, type II cuticular Hb4 |
| P78386 | IPI00032541 | 55802 | Keratin, type II cuticular Hb5 |
| NP_006112 | IPI00220327 | 66067 | Keratin, type II cytoskeletal 1 (Keratin-1, Cytokeratin 1; hair alpha protein) |
| Q7Z794 | IPI00376379 | 61688 | Keratin, type II cytoskeletal 1b |
| P35908 | IPI00021304 | 65865 | Keratin, type II cytoskeletal 2 epidermal |
| P13647 | IPI00009867 | 62447 | Keratin, type II cytoskeletal 5 |
| P02538 | IPI00300725 | 59914 | Keratin, type II cytoskeletal 6A |
| P48669 | IPI00293665 | 59868 | Keratin, type II cytoskeletal 6B |
| P48666 | IPI00479403 | 60069 | Keratin, type II cytoskeletal 6C |
| P48668 | IPI00299145 | 59894 | Keratin, type II cytoskeletal 6E |
| P05787 | IPI00005859/IPI00554648 | 53543 | Keratin, type II cytoskeletal 8 |
| O75037 | IPI00397809 | 182662 | Kinesin family member 21B |
| P01042-1 | IPI00032328 | 71945 | Kininogen-1 [Precursor], Splice Isoform 1 |
| P01042-2 | IPI00215894 | 47883 | Kininogen-1 [Precursor], Splice isoform 2 LMW |
| Q32MB2 | IPI00174775 | 42010 | KRT73 protein |
| P00338 | IPI00217966 | 36557 | L-lactate dehydrogenase A chain (LDH muscle subunit) |
| P07195 | IPI00219217 | 36507 | L-lactate dehydrogenase B chain |
| P14151 | IPI00218795 | 42187 | L-selectin [Precursor] |
| P02788 | IPI00298860 | 78182 | Lactotransferrin [Precursor] |
| Q04760 | IPI00220766 | 20588 | Lactoylglutathione lyase |
| Q9BS40 | IPI00106687 | 25769 | Latexin |
| HIT000042117 | IPI00022417 | 38583 | Leucine-rich alpha-2-glycoprotein precursor (highly similar) |
| P02750 | IPI00022417 | 38178 | Leucine-rich alpha-2-glycoprotein [Precursor] |
| O15506 | IPI00816779 | 38703 | Leukocyte antigen [Precursor] |
| P30740 | IPI00027444 | 42742 | Leukocyte elastase inhibitor |
| Q14847 | IPI00000861 | 29717 | LIM and SH3 domain protein 1 |
| P18428 | IPI00032311 | 53350 | Lipopolysaccharide-binding protein [Precursor] |
| P59827 | IPI00339367 | 60951 | Long palate, lung and nasal epithelium carcinoma-associated protein 4 |
| Q07954 | IPI00020557 | 504576 | Low-density lipoprotein receptor-related protein 1 [Precursor] |
| P51884 | IPI00020986 | 38429 | Lumican [Precursor] |
| Q8TC18 | IPI00290856 | 35213 | Lymphatic vessel endothelial hyaluronic acid receptor 1 [Precursor] |
| P13473 | IPI00009030 | 44961 | Lysosome-associated membrane glycoprotein 2 [Precursor], Splice Isoform 1 or 2 |
| P61626 | IPI00019038 | 16537 | Lysozyme C [Precursor] |
| P07333 | IPI00011218 | 107984 | Macrophage colony stimulating factor I receptor precursor |
| P14174 | IPI00293276 | 12345 | Macrophage migration inhibitory factor (glycosylation-inhibiting factor) |
| P40925 | IPI00291005 | 36295 | Malate dehydrogenase, cytoplasmic |
| P40926 | IPI00291006 | 35531 | Malate dehydrogenase, mitochondrial [Precursor] |
| O43451 | IPI00220143 | 209722 | Maltase-glucoamylase, intestinal (EC 3.2.1.20) (EC 3.2.1.3) |
| O00187-1 | IPI00294713 | 75686 | Mannan-binding lectin serine protease 2 [Precursor], MASP-2, Splice isoform 1 |
| P11226 | IPI00004373 | 26144 | Mannose-binding protein C [Precursor] (MBP) |
| P33908 | IPI00439446 | 72969 | Mannosyl-oligosaccharide 1,2-alpha-mannosidase IA |
| P10721 | IPI00022296 | 109865 | Mast/stem cell growth factor receptor [Precursor] |
| Q16853 | IPI00004457 | 84491 | Membrane copper amine oxidase |
| P01033 | IPI00032292 | 23171 | Metalloproteinase inhibitor 1 [Precursor] |
| P16035 | IPI00027166 | 24399 | Metalloproteinase inhibitor 2 [Precursor] |
| Q96KX8 | IPI00647704/IPI00473015 | 53392 | MGC27165 protein (81% and 82% sequence identity with Id488 and Id1118, respectively) |
| Q9BRV0 | IPI00061977 | 54154 | MGC27165 protein (81% and 85% sequence identity with Id1286 and Id 1118, respectively) |
| Q8N5K4 | IPI00166866 | 53376 | MGC27165 protein (82% and 85% sequence identity with Id1286 and Id488 respectively) |
| Q15555 | IPI00003420 | 37031 | Microtubule-associated protein RP/EB family member 2 (APC-binding protein EB2) (End-binding protein 2) (EB2) - Homo sapiens (Human) |
| P20774 | IPI00515092/IPI00025465 | 33922 | Mimecan [Precursor] (Osteoglycin) |
| P26038 | IPI00219365 | 67689 | Moesin |
| P08571 | IPI00029260 | 40076 | Monocyte differentiation antigen CD14 [Precursor] |
| Q6IBG9 | IPI00293590 | 34293 | Monoglyceride lipase isoform 1 or 2, MGLL protein |
| Q8TAX7 | IPI00152154 | 39171 | Mucin-7 [Precursor] |
| Q13201 | IPI00012269 | 138072 | Multimerin 1 [Precursor] |
| Q9H8L6 | IPI00015525 | 104417 | Multimerin 2 [Precursor] (EMILIN 3) |
| Q7Z7M0-1 | IPI00027310 | 254573 | Multiple epidermal growth factor-like domains 8 |
| Q9UNW1-1 | IPI00293748/IPI00028553 | 55051 | Multiple inositol polyphosphate phosphatase 1 [Precursor], splice isoform 1 (EC 3.1.3.62) (Inositol (1,3,4,5)-tetrakisphosphate 3-phosphatase) |
| Q71SW6 | IPI00165438 | 72066 | Muscle type neuropilin 1 |
| Q9H3K6 | IPI00301434 | 10117 | My016 protein( BolA-like), Splice Isoform 1 or 2 |
| P60201-1 | IPI00219661 | 29946 | Myelin proteolipid protein, Splice isoform 1 |
| Q9UL68 | IPI00007843 | 133861 | Myelin transcription factor 1-like |
| Q99972 | IPI00019190 | 56972 | Myocilin [Precursor] |
| P02144 | IPI00217493 | 17053 | Myoglobin |
| P05976 | IPI00216070 | 21014 | Myosin light chain 1, skeletal muscle isoform |
| P60660-2 | IPI00413922/IPI00027255 | 16830 | Myosin light polypeptide 6, Splice isoform 2 / Smooth muscle (Myosin light chain alkali 3) (Myosin light chain 3) (MLC-3) (LC17) |
| P19105 | IPI00033494/IPI00220573 | 19663 | Myosin regulatory light chain 2, nonsarcomeric |
| P24844 | IPI00030929/IPI00220278 | 19696 | Myosin regulatory light chain 2, smooth muscle isoform |
| P13535 | IPI00302329 | 222763 | Myosin-8 |
| P35579 | IPI00019502 | 226533 | Myosin-9 |
| P58546 | IPI00179589 | 12764 | Myotrophin |
| P20933 | IPI00026259 | 37194 | N(4)-(beta-N-acetylglucosaminyl)-L-asparaginase [Precursor] |
| Q9UJJ9 | IPI00000137 | 33974 | N-acetylglucosamine-1-phosphotransferase subunit gamma [Precursor] |
| Q96PD5 | IPI00163207/IPI00394992 | 62217 | N-acetylmuramoyl-L-alanine amidase [Precursor], Splice isoform 1 (EC 3.5.1.28) |
| Q59FL7 | IPI00411478/IPI00555628/IPI00220737 | 89176 | Neural cell adhesion molecule 1, 120 kDa isoform variant [Fragment] |
| Q14697 | IPI00011454 | 109438 | Neutral alpha-glucosidase AB [Precursor], Splice isoform 1 or 2 |
| P59665 | IPI00005721 | 10201 | Neutrophil defensin 1 [Precursor] |
| P80188 | IPI00299547 | 22588 | Neutrophil gelatinase-associated lipocalin [Precursor] |
| P14543 | IPI00026944 | 136489 | Nidogen [Precursor] |
| Q9GZT8 | IPI00604624 | 38984 | NIF3-like protein 1 |
| Q1WM23 | IPI00026260/IPI00604590 | 30137 | NM23-LV |
| P05204 | IPI00071125/IPI00217950 | 9261 | Nonhistone chromosomal protein HMG-17 |
| P61970 | IPI00009901 | 14478 | Nuclear transport factor 2 |
| P06748 | IPI00549248 | 32575 | Nucleophosmin, Splice isoform 1 or 2 |
| Q13232 | IPI00012315 | 19015 | Nucleoside diphosphate kinase 3 |
| P15531 | IPI00012048 | 17149 | Nucleoside diphosphate kinase A |
| P55209 | IPI00023860 | 45374 | Nucleosome assembly protein 1-like 1 |
| Q86UD1 | IPI00328703 | 30688 | OAF homolog |
| Q9H552 | IPI00017870 | 55152 | OTTHUMP00000021786 (BA13B9.3) |
| P16109 | IPI00295339 | 90845 | P-selectin [Precursor] |
| O95497 | IPI00030871 | 57024 | Pantetheinase [Precursor] |
| NP_000437 | IPI00218732 | 39731 | Paraoxonase 1 (PON1) |
| O00151 | IPI00010414 | 35941 | PDZ and LIM domain protein 1 |
| Q96S96 | IPI00163563 | 25431 | PEBP family protein [Precursor] |
| NP_066953 | IPI00549466/IPI00419585 | 18012 | Peptidyl-prolyl cis-trans isomerase A isoform 1 (Cyclophilin A) |
| P23284 | IPI00646304 | 22742 | Peptidyl-prolyl cis-trans isomerase B [Precursor] (Cyclophilin B) (EC 5.2.1.8) |
| Q15063-1 | IPI00007960 | 93314 | Periostin [Precursor] |
| Q06830 | IPI00000874 | 22110 | Peroxiredoxin 1 |
| P32119 | IPI00027350 | 21892 | Peroxiredoxin 2 |
| P30044 | IPI00024915 | 22026 | Peroxiredoxin 5, mitochondrial [Precursor] |
| P30041 | IPI00220301 | 24904 | Peroxiredoxin 6 (Antioxidant protein 2) |
| P04180 | IPI00022331 | 49578 | Phosphatidylcholine-sterol acyltransferase [Precursor] |
| P30086 | IPI00219446 | 20926 | Phosphatidylethanolamine-binding protein 1 |
| P80108 | IPI00299503 | 92375 | Phosphatidylinositol-glycan-specific phospholipase D 1 [Precursor] |
| P00558 | IPI00169383 | 44483 | Phosphoglycerate kinase 1 (EC 2.7.2.3) (Primer recognition protein 2) |
| P18669 | IPI00218570/IPI00453476 | 28673 | Phosphoglycerate mutase 1 (Phosphoglycerate mutase isozyme B) (PGAM-B) (BPG-dependent PGAM 1) [Homo sapiens]. |
| P55058-1 | IPI00022733/IPI00643034/IPI00217778 | 54740 | Phospholipid transfer protein [Precursor], Splice isoform 1 |
| P36955 | IPI00006114 | 46342 | Pigment epithelium-derived factor precursor |
| P48059 | IPI00007634 | 37251 | PINCH protein |
| P35237 | IPI00413451 | 42590 | Placental thrombin inhibitor |
| Q13835-1 | IPI00071509 | 82861 | Plakophilin-1 |
| P22352 | IPI00026199 | 25505 | Plasma glutathione peroxidase [Precursor] |
| P03952 | IPI00654888 | 71370 | Plasma kallikrein [Precursor] (Fletcher factor) |
| P05155 | IPI00291866 | 55154 | Plasma protease C1 inhibitor precursor |
| P02753 | IPI00022420 | 23010 | Plasma retinol-binding protein [Precursor] |
| P05154 | IPI00007221 | 45702 | Plasma serine protease inhibitor [Precursor] |
| P00747 | IPI00019580 | 90569 | Plasminogen [Precursor] |
| P13796 | IPI00010471 | 70158 | Plastin-2 |
| P02775 | IPI00022445 | 13894 | Platelet basic protein [Precursor] |
| Q96RF6 | IPI00157687 | 82536 | Platelet endothelial cell adhesion molecule [Precursor] |
| P02776 | IPI00022446/IPI00022295 | 10845 | Platelet factor 4 [Precursor] |
| P07359 | IPI00011255 | 68955 | Platelet glycoprotein Ib alpha chain [Precursor] |
| P13224 | IPI00464990 | 21718 | Platelet glycoprotein Ib beta chain [Precursor] |
| P14770 | IPI00027502 | 19046 | Platelet glycoprotein IX [Precursor] |
| P40197 | IPI00027410 | 60959 | Platelet glycoprotein V [Precursor] |
| P68402 | IPI00026546 | 25569 | Platelet-activating factor acetylhydrolase IB beta subunit |
| Q13093 | IPI00011588 | 50077 | Platelet-activating factor acetylhydrolase [Precursor] |
| P08567 | IPI00306311 | 40083 | Pleckstrin (Platelet p47 protein) |
| Q15365 | IPI00016610 | 37498 | Poly(rC)-binding protein 1 |
| P01833 | IPI00004573 | 83314 | Polymeric-immunoglobulin receptor [Precursor] |
| O60312 | IPI00514255 | 167689 | Potential phospholipid-transporting ATPase VA |
| Q8WY90 | IPI00556571 | 64912 | PP2500 (Multiple ankyrin repeats, single KH-domain protein, isoform 3) |
| XP_947165 | IPI00479116/IPI00738433/IPI00027504 | 60585 | PREDICTED: similar to Carboxypeptidase N subunit 2 precursor (Carboxypeptidase N polypeptide 2) (Carboxypeptidase N 83 kDa chain) (Carboxypeptidase N regulatory subunit) (Carboxypeptidase N large subunit) [Homo sapiens]. |
| P20742 | IPI00025426 | 163836 | Pregnancy zone protein precursor |
| Q9UHG3 | IPI00384280 | 56611 | Prenylcysteine oxidase [Precursor] |
| P01133 | IPI00000073 | 133946 | Pro-epidermal growth factor [Precursor] |
| Q8IZF3 | IPI00217512 | 83830 | Probable G-protein coupled receptor 115 |
| Q15113 | IPI00299738 | 47973 | Procollagen C-endopeptidase enhancer 1 [Precursor] |
| XP_499262 | IPI00454967 | 86384 | Procollagen, type VI, alpha 2, Predicted similarity |
| OTTHUMP00000077405 | IPI00373937 | 60541 | Product of gene OTTHUMG00000071331; novel; PREDICTED: similar to suprabasin [Canis familiaris] by BLAST |
| P07737 | IPI00216691 | 14923 | Profilin-1 |
| O75340 | IPI00025277 | 21869 | Programmed cell death protein 6 |
| P12273 | IPI00022974 | 16572 | Prolactin-inducible protein [Precursor] |
| Q96NZ9 | IPI00465255 | 17227 | Proline-rich acidic protein 1 or Uterine-specific proline-rich acidic protein |
| Q16378 | IPI00027019 | 15125 | Proline-rich protein 4 [Precursor] |
| P27918 | IPI00021364 | 51276 | Properdin [Precursor] (Factor P) |
| Q8NBP7-1 | IPI00387168 | 74372 | Proprotein convertase subtilisin/kexin type 9 [Precursor], Splice isoform 1 |
| P41222 | IPI00013179 | 21166 | Prostaglandin-H2 D-isomerase precursor |
| P25786 | IPI00016832 | 29556 | Proteasome subunit alpha type 1, Splice Isoform 1 or 2 |
| P25788-2 | IPI00171199 | 27516 | Proteasome subunit alpha type 3 |
| P25789 | IPI00299155 | 29484 | Proteasome subunit alpha type 4 |
| P60900 | IPI00029623 | 27399 | Proteasome subunit alpha type 6 |
| O14818-1 | IPI00024175 | 27887 | Proteasome subunit alpha type 7 |
| P20618 | IPI00025019 | 26489 | Proteasome subunit beta type 1 [Precursor] |
| P49721 | IPI00028006 | 22836 | Proteasome subunit beta type 2 |
| P49720 | IPI00028004 | 22949 | Proteasome subunit beta type 3 |
| P28072 | IPI00000811 | 25358 | Proteasome subunit beta type 6 [Precursor] |
| P30101 | IPI00025252 | 56783 | Protein disulfide-isomerase A3 [Precursor] |
| Q15084 | IPI00299571 | 48121 | Protein disulfide-isomerase A6 [Precursor] |
| P07237 | IPI00010796 | 57117 | Protein disulfide-isomerase [Precursor] |
| Q9HCY8 | IPI00010214 | 11662 | Protein S100-A14 |
| P31151 | IPI00219806 | 11326 | Protein S100-A7 |
| Q96DA0 | IPI00060800 | 19600 | Protein UNQ773/PRO1567 [Precursor] |
| Q9UK55 | IPI00007199 | 50707 | Protein Z-dependent protease inhibitor [Precursor] |
| Q08188 | IPI00300376 | 76632 | Protein-glutamine gamma-glutamyltransferase E [Precursor] |
| P22735 | IPI00305622 | 89787 | Protein-glutamine gamma-glutamyltransferase K |
| Q92954-1 | IPI00024825 | 151077 | Proteoglycan-4 [Precursor], splice isoform 1, 3, or 6 (Megakaryocyte stimulating factor) |
| P00734 | IPI00019568/IPI00006618 | 70037 | Prothrombin [Precursor] (EC 3.4.21.5) |
| P11309 | IPI00005014 | 35686 | Proto-oncogene serine/threonine-protein kinase Pim-1 |
| P00491 | IPI00017672 | 32118 | Purine nucleoside phosphorylase (EC 2.4.2.1) |
| P14618-2 | IPI00220644 | 57931 | Pyruvate kinase, isozymes M1/M2 (Pyruvate kinase muscle isozyme, Cytosolic thyroid hormone-binding protein (CTHBP) (THBP1)), Splice isoform 2 (M1) |
| Q2NKL2 | IPI00719195 | 140364 | PZP protein |
| P31150 | IPI00010154 | 50583 | Rab GDP dissociation inhibitor alpha (Rab GDI alpha) (GDI-1) (XAP-4) (Oligophrenin 2) |
| P50395 | IPI00031461 | 50663 | Rab GDP dissociation inhibitor beta |
| Q15404 | IPI00017256 | 31409 | Ras suppressor protein 1 isoform 1 |
| P15153 | IPI00010270 | 21429 | Ras-related C3 botulinum toxin substrate 2 |
| O95916 | IPI00001352 | 21383 | Ras-related C3 botulinum toxin substrate 4, Putative |
| Q9H0U4 | IPI00008964 | 22171 | Ras-related protein Rab-1B |
| O00194 | IPI00010491 | 24608 | Ras-related protein Rab-27B |
| P61019 | IPI00031169 | 23546 | Ras-related protein Rab-2A |
| Q15286 | IPI00300096 | 23025 | Ras-related protein Rab-35 |
| P51149 | IPI00016342 | 23490 | Ras-related protein Rab-7 |
| P61224 | IPI00015148 | 20825 | Ras-related protein Rap-1b |
| Q12913 | IPI00290328 | 145927 | Receptor-type tyrosine-protein phosphatase eta [Precursor] |
| P10586 | IPI00107831 | 211845 | Receptor-type tyrosine-protein phosphatase F [Precursor] |
| P23470 | IPI00011651 | 162059 | Receptor-type tyrosine-protein phosphatase gamma [Precursor] |
| Q13332 | IPI00289831 | 217095 | Receptor-type tyrosine-protein phosphatase S [Precursor], isoform 1, 2, 3, or 4; others possible (not isoform 5) |
| Q99969 | IPI00019176 | 18618 | Retinoic acid receptor responder protein 2 [Precursor] |
| P52565 | IPI00003815 | 23207 | Rho GDP-dissociation inhibitor 1 |
| P52566 | IPI00003817 | 22988 | Rho GDP-dissociation inhibitor 2 |
| P08134 | IPI00027434 | 22006 | Rho-related GTP-binding protein RhoC |
| P34096 | IPI00029699 | 16840 | Ribonuclease 4 [Precursor] |
| P07998 | IPI00014048 | 17644 | Ribonuclease pancreatic [Precursor] |
| P31153 | IPI00010157 | 43661 | S-adenosylmethionine synthetase gamma form |
| P26447 | IPI00032313 | 11729 | S100 calcium-binding protein A4 |
| Q4F965 | IPI00413626 | 30703 | Sad1 and UNC84 domain containing 1 |
| Q07901 | IPI00513892/IPI00104074 | 125437 | Scavenger receptor cysteine-rich type 1 protein M130 [Precursor] (CD163) isoform 1, 2, 3 or 4 |
| Q13103 | IPI00011832 | 24338 | Secreted phosphoprotein 24 [Precursor] |
| P49908 | IPI00029061 | 42706 | Selenoprotein P [Precursor] |
| Q16181 | IPI00033025 | 50680 | Septin-7 |
| Q15173 | IPI00014980 | 57393 | Serine/threonine protein phosphatase 2A, 56 kDa regulatory subunit, beta isoform, Splice isoform 1 or 2 |
| Q96PY6 | IPI00044749 | 142829 | Serine/threonine-protein kinase Nek1, Splice isoform 1 or 2 |
| P02787 | IPI00022463 | 77050 | Serotransferrin [Precursor] |
| Q86U17 | IPI00333828 | 46989 | Serpin A11 [Precursor] |
| Q96P63 | IPI00033583 | 46276 | Serpin B12 |
| P29508 | IPI00022204 | 44565 | Serpin B3 |
| P02768 | IPI00022434/IPI00745872 | 69367 | Serum albumin [Precursor], splice isoform 1 |
| P02735 | IPI00552578 | 13532 | Serum amyloid A protein [Precursor] |
| NP_110381 | IPI00006146 | 13508 | Serum amyloid A-2 protein |
| P35542 | IPI00019399 | 14807 | Serum amyloid A-4 protein [Precursor] |
| P02743 | IPI00022391 | 25387 | Serum amyloid P-component [Precursor] |
| O95810 | IPI00005809 | 47173 | Serum deprivation response |
| P27169 | IPI00218732 | 39618 | Serum paraoxonase/arylesterase 1 |
| Q15166 | IPI00299778 | 39608 | Serum paraoxonase/lactonase 3 |
| P04278 | IPI00023019 | 43779 | Sex hormone-binding globulin precursor, Splice Isoform 1 |
| O75368 | IPI00025318 | 12774 | SH3 domain-binding glutamic acid-rich-like protein |
| Q9UJC5 | IPI00412272 | 12326 | SH3 domain-binding glutamic acid-rich-like protein 2 |
| Q9H299 | IPI00010402 | 10438 | SH3 domain-binding glutamic acid-rich-like protein 3 |
| Q9Y286 | IPI00004288 | 51143 | Sialic acid binding Ig-like lectin 7 [Precursor], Splice Isoform 1, 2, 3, or 4 |
| Q9UIU0 | IPI00470535 | 125308 | Similar to L-type calcium channel alpha2/delta subunit [Rattus norvegicus] by BLAST; novel; similar to Dihydropyridine receptor alpha 2 subunit - Homo sapiens (Human). |
| HIT000022175 | IPI00641034 | 54350 | SNC66 protein, Similar |
| Q15465 | IPI00017480 | 49607 | Sonic hedgehog protein [Precursor] |
| P09486 | IPI00014572 | 34632 | SPARC [Precursor] |
| Q14515 | IPI00296777 | 75216 | SPARC-like protein 1 precursor |
| P48594 | IPI00010303 | 44854 | Squamous cell carcinoma antigen 2 |
| P02808 | IPI00022990 | 7305 | Statherin [Precursor] |
| P38646 | IPI00007765 | 73680 | Stress-70 protein, mitochondrial [Precursor] |
| O00391 | IPI00003590 | 82578 | Sulfhydryl oxidase 1 [Precursor] (Quiescin Q6) |
| P00441 | IPI00218733 | 15804 | Superoxide dismutase [Cu-Zn] |
| P04179 | IPI00022314 | 24722 | Superoxide dismutase [Mn], mitochondrial [Precursor] |
| Q9Y490 | IPI00298994 | 269767 | Talin-1 |
| P24821-1 | IPI00031008 | 240867 | Tenascin [Precursor] |
| P22105-1 | IPI00025276 | 464458 | Tenascin-X [Precursor], splice isoform 1 |
| P05452 | IPI00009028 | 22567 | Tetranectin [Precursor] |
| P10599 | IPI00216298 | 11606 | Thioredoxin |
| Q9BRA2 | IPI00646689 | 13941 | Thioredoxin-like protein 5 |
| P07996 | IPI00296099 | 129413 | Thrombospondin-1 [Precursor] |
| P35443 | IPI00328550 | 105869 | Thrombospondin-4 [Precursor] |
| P62328 | IPI00220828/IPI00180240 | 4921 | Thymosin beta-4 |
| P05543 | IPI00292946 | 46324 | Thyroxine-binding globulin [Precursor] |
| P10646-1 | IPI00021834 | 35015 | Tissue factor pathway inhibitor [Precursor] |
| Q8WZ42 | IPI00745683/IPI00006543/IPI00179357/  IPI00023283 | 3816219 | Titin (EC 2.7.11.1), splice isoforms 1, 2, 4, 7, 8 |
| P37837 | IPI00550488 | 37540 | Transaldolase (EC 2.2.1.2) |
| Q9UGU0 | IPI00159322 | 211800 | Transcription factor 20, Splice isoform 1 or 2 |
| P01137 | IPI00000075 | 44341 | Transforming growth factor beta-1 [Precursor] |
| Q15582 | IPI00018219 | 74681 | Transforming growth factor-beta induced protein IG-H3 [Precursor] |
| P37802 | IPI00550363 | 22260 | Transgelin-2 |
| P02766 | IPI00022432 | 15887 | Transthyretin [Precursor] |
| Q96HR3 | IPI00063213 | 20277 | TRAP/Mediator complex component TRAP25 |
| HIT000194072 | IPI00465028/IPI00451401/IPI00383071 | 30536 | Triosephosphate isomerase (EC 5.3.1.1) (TIM) (Triose-phosphate isomerase) |
| Q7Z6L8 | IPI00384369/IPI00000230 | 28386 | Tropomyosin 1 alpha chain, isoform 6 |
| P09493-2 | IPI00216134 | 26549 | Tropomyosin 1 alpha chain, Splice Isoform 2 |
| P09493-3 | IPI00216135 | 32876 | Tropomyosin 1 alpha chain, Splice Isoform 3 |
| P06753-2 | IPI00479185/IPI00216975/IPI00218319/  IPI00183968/IPI00178083 | 28902 | Tropomyosin alpha 3 chain, splice isoform 2 |
| P67936-1 | IPI00010779 | 28391 | Tropomyosin alpha 4 chain (Tropomyosin 4) (TM30p1), splice isoform 1 |
| P07951-1 | IPI00013991 | 32851 | Tropomyosin beta chain, Splice isoform 1 |
| P07477 | IPI00011694 | 26558 | Trypsin I [Precursor] |
| P07478 | IPI00011695 | 26488 | Trypsin-2 [Precursor] |
| Q8WUA8 | IPI00641368 | 37807 | Tsukushi [Precursor] |
| Q8N532 | IPI00166768/IPI00387144 | 36649 | TUBA6 protein |
| P68366 | IPI00007750 | 49925 | Tubulin alpha-1 chain (Alpha-tubulin 1) (Testis-specific alpha- tubulin) (Tubulin H2-alpha) - Homo sapiens (Human). |
| Q8WU19 | IPI00387144/IPI00218343 | 37218 | Tubulin alpha-ubiquitous chain (K-ALPHA-1 protein) (Alpha-tubulin ubiquitous) (Tubulin K- alpha-1) (and possibly Tubulin alpha-6 chain) |
| Q9H4B7 | IPI00006510 | 50327 | Tubulin beta-1 chain |
| P68371 | IPI00007752 | 49831 | Tubulin beta-2 (?) chain |
| P07437 | IPI00011654 | 49671 | Tubulin beta-2 chain |
| O75347 | IPI00217236 | 12724 | Tubulin-specific chaperone A |
| Q9Y274 | IPI00184851 | 38214 | Type 2 lactosamine alpha-2,3-sialyltransferase |
| Q7Z3Z0 | IPI00375911 | 49318 | Type I inner root sheath specific keratin 25 irs1 |
| Q7Z3Y8 | IPI00328103 | 49823 | Type I inner root sheath specific keratin 25 irs3 |
| NP_002945 | IPI00179330 | 17965 | Ubiquitin and ribosomal protein S27a precursor |
| P62837 | IPI00019932 | 16735 | Ubiquitin-conjugating enzyme E2 D2 |
| P68036 | IPI00021347 | 17862 | Ubiquitin-conjugating enzyme E2 L3 |
| P61088 | IPI00003949 | 17138 | Ubiquitin-conjugating enzyme E2 N |
| Q13404 | IPI00019599 | 25797 | Ubiquitin-conjugating enzyme E2 variant 1, Splice Isoform 1 Of |
| P30085 | IPI00219953 | 22222 | UMP-CMP kinase |
| Q86UX7 | IPI00216699 | 75430 | Unc-112 related protein 2, Splice isoform 1 or 2 |
| Q9P1F3 | IPI00514113/IPI00374316 | 9056 | Uncharacterized protein C6orf115 (Chromosome 6 open reading frame 115) |
| P07911 | IPI00013945 | 69761 | Uromodulin [Precursor] |
| Q5NV81 | IPI00045547/IPI00385254 | 10284 | V1-16 protein [Fragment] |
| Q5NV68 | IPI00514517 | 11238 | V4-1 protein [Fragment] |
| P19320 | IPI00018136 | 81276 | Vascular cell adhesion protein 1 [Precursor], Splice Isoform 1 or 2 |
| P33151 | IPI00012792 | 87516 | Vascular endothelial-cadherin [Precursor] |
| Q6UXL4 | IPI00395488 | 71713 | Vasorin (slit-like 2) |
| Q12907 | IPI00009950 | 40229 | Vesicular integral-membrane protein VIP36 [Precursor] |
| O95978 | IPI00383957 | 17304 | VH1 protein [Precursor] [Fragment] |
| P08670 | IPI00418471/IPI00013164 | 53521 | Vimentin |
| P18206 | IPI00291175 | 116591 | Vinculin isoform VCL, splice isoform 1 or 2 |
| ENSP00000273951 | IPI00555812/IPI00742696 | 52918 | Vitamin D-binding protein precursor |
| P02774 | IPI00555812 | 52964 | Vitamin D-binding protein precursor |
| P04070 | IPI00021817 | 52071 | Vitamin K-dependent protein C precursor (EC 3.4.21.69) (Autoprothrombin IIA) (Anticoagulant protein C) (Blood coagulation factor XIV) |
| P07225 | IPI00294004 | 75123 | Vitamin K-dependent protein S [Precursor] |
| P22891 | IPI00027843 | 44744 | Vitamin K-dependent protein Z [Precursor], Splice Isoform 1 or 2 |
| P04004 | IPI00298971 | 54306 | Vitronectin [Precursor] |
| Q13303 | IPI00021088 | 41000 | Voltage-gated potassium channel beta-2 subunit, Splice Isoform 1 or 2 |
| P31025 | IPI00009650 | 19250 | Von Ebner's gland protein [Precursor] |
| P04275 | IPI00023014 | 309299 | Von Willebrand factor [Precursor] |
| Q96L37 | IPI00449028 | 153633 | Von Willebrand factor-cleaving protease [Precursor] (ADAMTS-13) |
| P12955 | IPI00257882 | 54417 | Xaa-Pro dipeptidase |
| Q9HBT8 | IPI00010163 | 60175 | Zinc finger protein 286 |
| O60844 | IPI00029647 | 18177 | Zymogen granule membrane protein 16 [Precursor] |
| Q15942 | IPI00020513 | 61278 | Zyxin |
